# Earth-abundant Li-ion cathode materials with nanoengineered microstructures

**DOI:** 10.1038/s41565-024-01787-y

**Published:** 2024-09-19

**Authors:** Han-Ming Hau, Tara Mishra, Colin Ophus, Tzu-Yang Huang, Karen Bustilo, Yingzhi Sun, Xiaochen Yang, Tucker Holstun, Xinye Zhao, Shilong Wang, Yang Ha, Gi-Hyeok Lee, Chengyu Song, John Turner, Jianming Bai, Lu Ma, Ke Chen, Feng Wang, Wanli Yang, Bryan D. McCloskey, Zijian Cai, Gerbrand Ceder

**Affiliations:** 1 Department of Materials Science and Engineering, University of California Berkeley, Berkeley, CA USA; 2 Materials Sciences Division, Lawrence Berkeley National Laboratory, Berkeley, CA USA; 3 National Center for Electron Microscopy, Molecular Foundry, Lawrence Berkeley National Laboratory, Berkeley, CA USA; 4 Department of Chemical and Biomolecular Engineering, University of California Berkeley, Berkeley, CA USA; 5 Energy Storage and Distributed Resources Division, Lawrence Berkeley National Laboratory, Berkeley, CA USA; 6 Advanced Light Source, Lawrence Berkeley National Laboratory, Berkeley, CA USA; 7 National Synchrotron Light Source II, Brookhaven National Laboratory, Upton, NY USA; 8 Applied Materials Division, Argonne National Laboratory, Lemont, IL USA

**Keywords:** Batteries, Batteries

## Abstract

Manganese-based materials have tremendous potential to become the next-generation lithium-ion cathode as they are Earth abundant, low cost and stable. Here we show how the mobility of manganese cations can be used to obtain a unique nanosized microstructure in large-particle-sized cathode materials with enhanced electrochemical properties. By combining atomic-resolution scanning transmission electron microscopy, four-dimensional scanning electron nanodiffraction and in situ X-ray diffraction, we show that when a partially delithiated, high-manganese-content, disordered rocksalt cathode is slightly heated, it forms a nanomosaic of partially ordered spinel domains of 3–7 nm in size, which impinge on each other at antiphase boundaries. The short coherence length of these domains removes the detrimental two-phase lithiation reaction present near 3 V in a regular spinel and turns it into a solid solution. This nanodomain structure enables good rate performance and delivers 200 mAh g^−1^ discharge capacity in a (partially) disordered material with an average primary particle size of ∼5 µm. The work not only expands the synthesis strategies available for developing high-performance Earth-abundant manganese-based cathodes but also offers structural insights into the ability to nanoengineer spinel-like phases.

## Abstract (web summary)

By inducing a transformation in a manganese-rich cation-disordered rocksalt, partially ordered spinels with nanomosaic domains of 3–7 nm in size can be obtained, which exhibit high energy density and rate capability at an average particle size of 3–5 µm.

## Main

Rechargeable lithium-ion batteries have played an important role in technological advancement by providing high energy density and long cycle-life energy storage. Progress in lithium-ion batteries has been driven by well-ordered structures such as layered and spinel compounds, which rely on the stability of nickel and cobalt in octahedral sites to achieve stable cycling performance^1–8^. With higher energy demands arising from the need for electric vehicles with extended range and personal electronics that can operate for longer times, the low abundance of nickel and cobalt, and ethical concerns related to cobalt mining, may increase the cathode cost and place a large burden on the supply chain^9,10^. Other, more Earth-abundant transition metals, such as chromium, manganese, iron and copper, lack the intrinsic site stability of nickel and cobalt and require novel crystal and microstructure engineering to be useful as lithium-ion energy storage materials. Manganese-based cathodes would be particularly attractive because of the low toxicity and cost of manganese minerals, the ideal voltage range of the Mn^3+^/Mn^4+^ redox couple and the high stability of the charged Mn^4+^ state which leads to better safety and energy density gains when integrating cells at the pack level.

In the last decade, lithium-excess cation-disordered rocksalt (DRX) materials have demonstrated the potential for high energy density with Earth-abundant elements, including manganese, chromium and titanium^11–21^. However, the practical use of DRX materials requires milling to nanosized particles to achieve reasonable rate capability. The high surface area of nanoscale materials aggravates parasitic reactions in batteries and leads to lower-density electrodes. Being able to utilize a larger particle size while maintaining high performance is therefore important for DRX to become commercially viable^22,23^.

In this work, we demonstrate through in-depth atomistic characterization with atomic-resolution STEM^24^ and scanning electron nanodiffraction (SEND)^25^ that a high-manganese-content DRX can be transformed into a unique nanostructured material in which nanosized, partially disordered spinel domains with a coherence length of 3–7 nm are separated by antiphase boundaries. This microstructure decouples the (nano) length scale, at which lithiation thermodynamics and the voltage curve are determined, from the particle size, keeping it large enough to achieve good cycling and practical electrode manufacturing. The small coherence length of the nanosized spinel-like domains modifies the two-phase reaction of a regular spinel into a solid solution, thereby removing a major obstacle to achieving high energy density and rate capability in manganese-based DRX cathode materials at the large particle scale. We refer to this structure as δ-phase, and show that it can be achieved in a two-step process involving chemical delithiation followed by low-temperature heating—in contrast to the extended (>3 weeks) of electrochemical cycling previously needed to improve the performance of high-manganese-content DRX materials^26–29^.

## Structural characterization of DRX and δ-phase materials

**Fig. 1:**
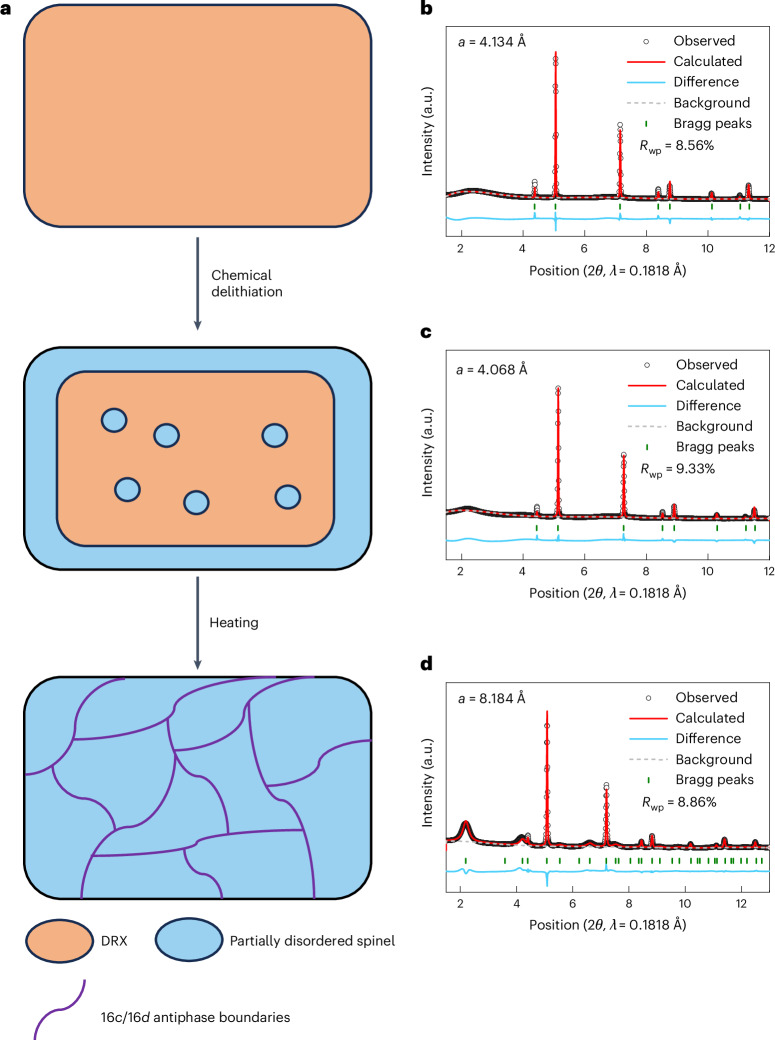
Phase evolution of L12M65. **a**, Schematic of the structural evolution of L12M65 with each synthesis step. **b**–**d**, Synchrotron XRD patterns of L12M65 (**b**), L07M65-D (**c**) and L07M65-DH (**d**). Source data

We chose Li_1.2_Mn_0.65_Ti_0.15_O_1.9_F_0.1_ (L12M65) as a starting material for the study because its high manganese content and low titanium content have been shown to lead to a transformation that improves performance upon electrochemical cycling^26,28^. The 20% lithium excess allows for good lithium-ion percolation^11,30^. Figure 1a conceptualizes the structural changes anticipated through each synthesis step. The basic DRX L12M65 is synthesized through a standard solid-state method^26^. The synchrotron X-ray diffraction (XRD) pattern (Fig. 1b) confirms that the as-synthesized L12M65 consists of pure DRX without any detectable impurities. Rietveld refinement based on the rocksalt structure (*Fm–3m*) gives a lattice parameter of 4.133 Å (Supplementary Table 1). Particle morphology measured by scanning electron microscopy (SEM) for the as-synthesized samples indicates a primary particle size of 20 µm for L12M65 (Supplementary Fig. 1a,c). Delithiation to Li_0.7_Mn_0.65_Ti_0.15_O_1.9_F_0.1_ (L07M65-D) was achieved by applying a solution of 0.1 M NO_2_BF_4_ at 45 °C for 2 days. XRD of the resulting sample (Fig. 1c) shows peak shifts toward higher angles, consistent with delithiation. Rietveld refinement based on the rocksalt structure (*Fm–3m*) for L07M65-D gives a refined lattice parameter of 4.067 Å (Supplementary Table 2). The increase in (111) peak intensity at 2.13° may indicate the emergence of the δ-phase. After heat treatment at 200 °C for 2 h, L07M65-DH shows a further increase in (111) peak intensity (Fig. 1d), and SEM indicates an average particle size of 12 µm (Supplementary Fig. 1b,c). The broadening of the (111), (311) and (333) peaks, which are consistent with spinel-type ordering, indicates a short coherence length of this cation ordering. Rietveld refinement modelled on a spinel structure (*Fd–3m*) with selective peak broadening applied to Bragg peaks with an odd *l* index leads to a good fit with *R*_wp_ = 8.86% and a refined lattice parameter of 8.211 Å (Supplementary Table 3)^31^. The 8a lithium occupancy is estimated from the capacity delivered in the 4 V region during the first discharge of L07M65-DH. Inductively coupled plasma mass spectrometry results, provided in Supplementary Table 4, confirm that the metal ratios in L12M65 are close to the target compositions and the delithiation level is close to the expected values for L07M65-D and L07M65-DH.

**Fig. 2:**
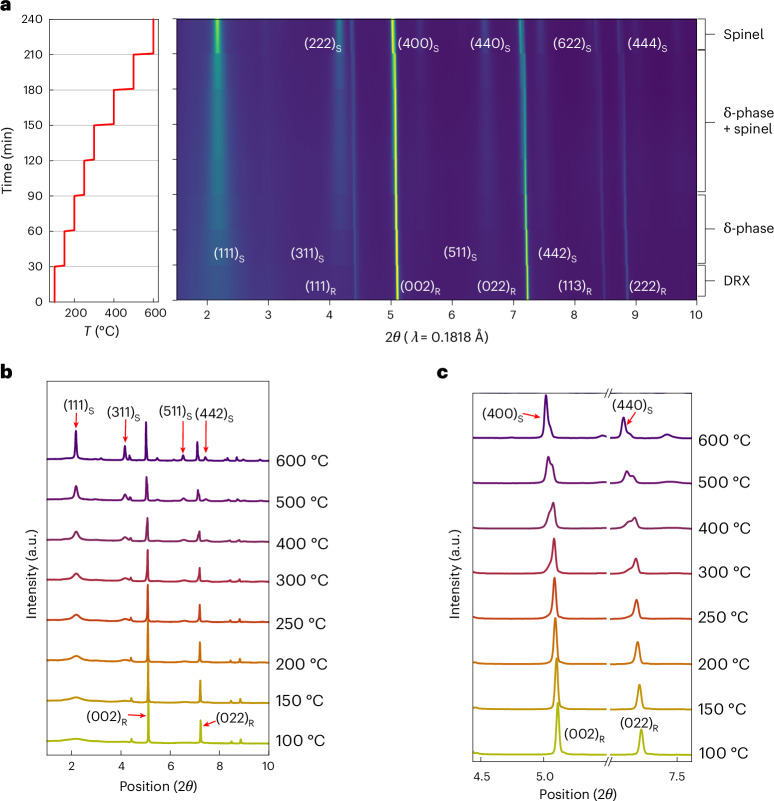
In situ heating XRD of L07M65-D. **a**, Temperature–time profile (left) and intensity map (right) highlighting the evolution of the Bragg peaks. A phase sequence of DRX to δ to spinel can be observed. **b**, Selected XRD patterns of L07M65-D when heated from 100 °C to 600 °C. Rocksalt (spinel) peaks are marked with subscript R (S). **c**, Zoomed-in XRD pattern between 4.5° and 7.5° showing the change from rocksalt (002) and (022) peaks to spinel (400) and (440) peaks between 100 °C and 600 °C. Source data

We further explored the changes that can be induced in the partially delithiated L07M65-D by in situ XRD when heating the sample from 100 °C to 600 °C (Fig. 2a). The selected XRD patterns of L07M65-D after heating at each temperature for 20 min are shown in Fig. 2b. At 100 °C, L07M65-D shows dominant (002) and (022) peaks that result from the DRX structure. Between 150 °C and 200 °C, broad features emerge for diffraction peaks with odd *l* indexes (for example, the (111) peak at 2.13° and the (311) peak at 4.21°). In contrast, the enlarged views of the (002) and (022) rocksalt peaks in Fig. 2c show that these do not broaden, indicating that the grain size over which the anion lattice extends remains unchanged. Upon heating from 250 °C to 500 °C, the diffraction peaks with odd *l* indexes sharpen and increase in intensity (Fig. 2a,b). In addition, the (002) and (022) peaks from the δ-phase at ~5.11° and ~7.23° in Fig. 2c split, indicating the formation of a new phase with new peaks at 5.04° and 7.14°. In fact, the peak splitting can be observed for all diffraction peaks with even *l* indexes. Above 500 °C, all peaks become sharper with increased peak intensity (Fig. 2b). Given the spinel-type cation ordering in the δ-phase, the new phase is likely to be a spinel with a larger lattice parameter, possibly due to the reduction induced by heating. For samples that were heated at 500 °C and 600 °C after delithiation, the voltage profile shows an increase in the capacity near 4 V and a decrease near 3 V, more resembling that of an ordered spinel phase. These changes of the voltage profile with elevated temperature annealing would indicate that at too high a temperature the δ-phase gradually transforms to a well-ordered spinel (Supplementary Fig. 2a,b). Hence, we focus on the 200 °C annealed sample.

## Atomic-scale characterization of the δ-phase

**Fig. 3:**
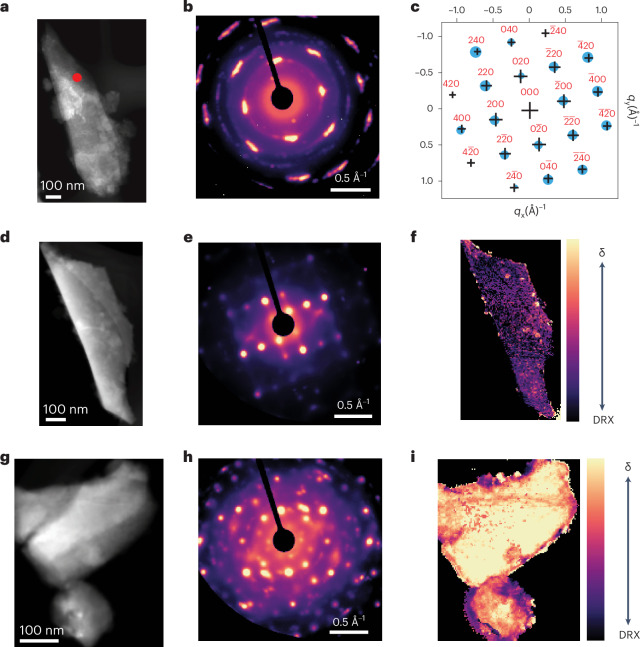
SEND characterization of L12M65, L07M65-D and L07M65-DH. **a**, HAADF image of L12M65. **b**, Mean SEND pattern of an L12M65 particle. **c**, Indexed diffraction pattern where the diffraction spots observed experimentally are marked as blue dots and the simulation as black crosses from the region marked by a red dot in **a**. **d**, HAADF image of L07M65-D. **e**, SEND pattern of an L07M65-D particle. **f**, Colour-coded spatial distribution of the δ-phase DRX. **g**, HAADF image of L07M65-DH. **h**, SEND pattern an L07M65-DH particle. **i**, Colour-coded spatial distribution of the δ-phase.

To obtain a more detailed understanding of the structural evolution of the sample upon chemical delithiation and heating, we conducted four-dimensional SEND on L12M65 (pristine), L07M65-D (after delithiation) and L07M65-DH (after delithiation and heat treatment at 200 °C). Figure 3a shows the high-angle annular dark-field (HAADF) image of L12M65 on which SEND patterns were collected. The mean of the diffraction patterns from this particle is shown in Fig. 3b. The sharp diffraction spots suggest a high degree of crystallinity of the pristine DRX particle. The mean diffraction pattern shows arcs in the diffraction spots, indicating a small in-plane rotational variation in this largely single-crystal particle. A selected diffraction pattern from the SEND dataset from region marked as a red dot in Fig. 3a, all of which can be indexed to a rocksalt structure, is shown and indexed in Fig. 3c, suggesting the absence of any other phase. To further confirm the absence of any other phase, the intensity from the diffraction peaks across the particle is radially integrated and overlaid with the powder pattern for the *Fm–3m* space group in Supplementary Fig. 3.

The mean SEND patterns of the chemically delithiated L07M65-D particle (Fig. 3d) are shown in Fig. 3e. Diffraction patterns from selected points and indexed based on the structural information obtained using XRD information of the same samples (Supplementary Tables 1–3) are provided in Supplementary Fig. 4. By integrating the intensity of the diffraction reflections unique to the δ-phase from each SEND pattern and normalizing it with the full acquired scattering range (from 0.175 A^−1^ to 1.2 A^−1^) to account for thickness, we can map the spatial extent of the δ-phase. The ranges of radial scattering vectors are shown in Supplementary Fig. 5 and are detailed in the Methods. The effect of thickness normalization is shown in Supplementary Fig. 6, which shows a control sample of uniform thickness made using a focused ion beam. Detailed explanation of the thickness normalization is given in Supplementary Note 1. Figure 3f shows the spatial distribution of the δ-phase. The δ-phase intensity is higher in the surface layer (50 nm) than in the bulk. The results indicate that much of the DRX-to-δ transformation initiates from the surface during the delithiation process. However, even in the bulk region there exist pockets of substantial DRX-to-δ transformation.

We undertook a similar procedure to map the δ-phase extent in a L07M65-DH sample that was chemically delithiated and subsequently heated at 200 °C for 2 h. The STEM-HAADF image of the particle is shown in Fig. 3g and the mean SEND diffraction pattern in Fig. 3h. Indexed diffraction patterns are provided in Supplementary Fig. 7. Figure 3i shows that the entire particle has uniformly transformed into the δ-phase after heating. The δ-phase signal has much higher intensity after heating (Fig. 3i), suggesting that the heat treatment is vital for the complete formation of the δ-phase.

It is important to note that in the radial integration patterns shown in Supplementary Figs. 3, 5 and 8, the crystal planes that are normal to the electron beam will not be represented in the transmission electron microscopy (TEM) diffraction data, which can explain the absence of some of the simulated peaks in the experimental radially integrated diffraction pattern. Additionally, the kinematical diffraction approximation with which the DRX peaks are simulated does not capture the dynamic electron scattering occurring due to the thickness of the sample, thereby leading to mismatch in the intensity between the simulated and experimentally observed diffraction patterns^32^. Furthermore, this kinematical approximation in the simulations would also lead to the absence of certain peaks observed experimentally due to multiple scattering in thick samples in Supplementary Figs. 4 and 7.

**Fig. 4:**
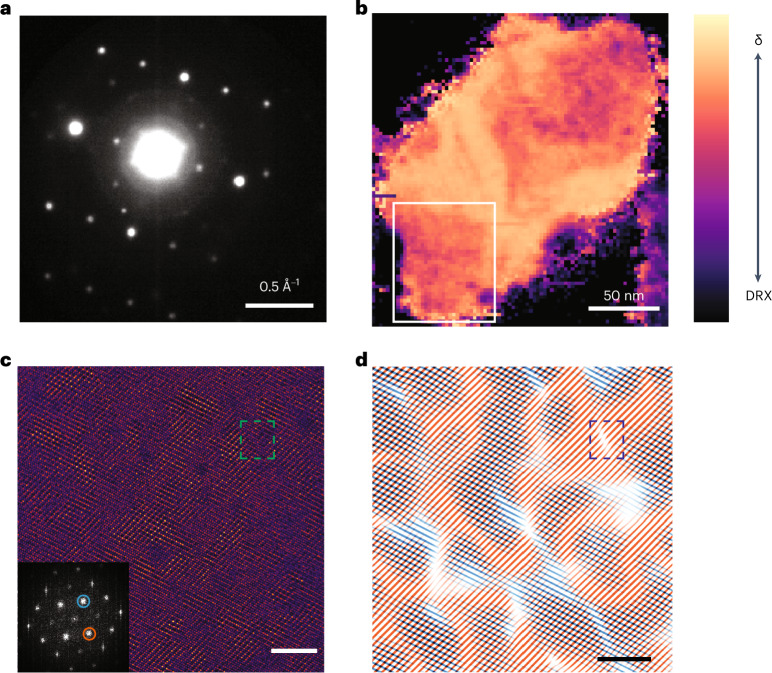
Multimodal characterization of L07M65-DH. **a**, Mean SEND pattern of L07M65-DH. **b**, Integrated spatial distribution of the δ-phase from the SEND patterns collected from the particle. **c**, Atomic-resolution HAADF-STEM image collected from the region marked with a white box in **b**, with the amplitude of its Fourier transform shown in the inset. Scale bar, 5 nm. **d**, Cation ordering from Bragg filtering applied to the frequency components marked on the Fourier transform shown in the inset of **c**. The blue dashed box shows a translation in the spinel-like lattice fringes representative of an antiphase boundary. Scale bar, 5 nm.

To investigate the cation ordering in the δ-phase in detail, we augmented SEND with atomic-resolution HAADF imaging on a particle from the L07M65-DH sample. To study the lithium distribution following chemical delithiation, electron energy loss spectroscopy (EELS) of L07M65-DH was also measured on the same particle. The homogeneous compositional mapping of the lithium, manganese and titanium throughout the particle, as shown in Supplementary Fig. 9, confirms that there is no preferential delithiation during the synthesis, subsequent delithiation and heating. Figure 4a shows the mean SEND pattern from this particle. Integration of the diffraction peaks unique to the δ-phase and normalized with the full acquired scattering range (from 0.175 A^−1^ to 1.2 A^−1^) from the SEND patterns from the particle is shown in Fig. 4b, confirming that the entire particle transforms into the δ-phase. The noise-filtered atomic-resolution image collected from the region marked with a white square in Fig. 4b is shown in Fig. 4c; the raw micrograph is shown in Supplementary Fig. 10a. The fast Fourier transform of the HAADF-STEM micrograph is shown as an inset in Fig. 4c. An inverse Fourier transform of the spinel-like peaks marked with a red and blue circle in the inset of Fig. 4c is shown in Fig. 4d. The fringes of the filtered image in Fig. 4d are colour-coded based on the isolated frequency component used to obtain them, as marked in the inset of Fig. 4c. For visual clarity, spinel fringes obtained by filtering the different frequency components corresponding to the spinel phase are plotted separately in Supplementary Fig. 10. Details of the filtering method are provided in the Methods. A prevalence of antiphase boundaries, where one variant of the spinel ordering meets another, can be observed. Once such antiphase boundary is shown in the blue dashed box in the top right of Fig. 4d. Furthermore, in between the spinel domains, thin, disordered regions are also observed. The domain size of the δ-phase is estimated to range from 3 to 7 nm by counting the average number of lattice fringes between the antiphase boundaries, and agrees well with the calculated coherence length obtained by applying the Scherrer equation to the (111) XRD peak in Fig. 1d^33^.

## Electrochemical performance of the δ-phase

**Fig. 5:**
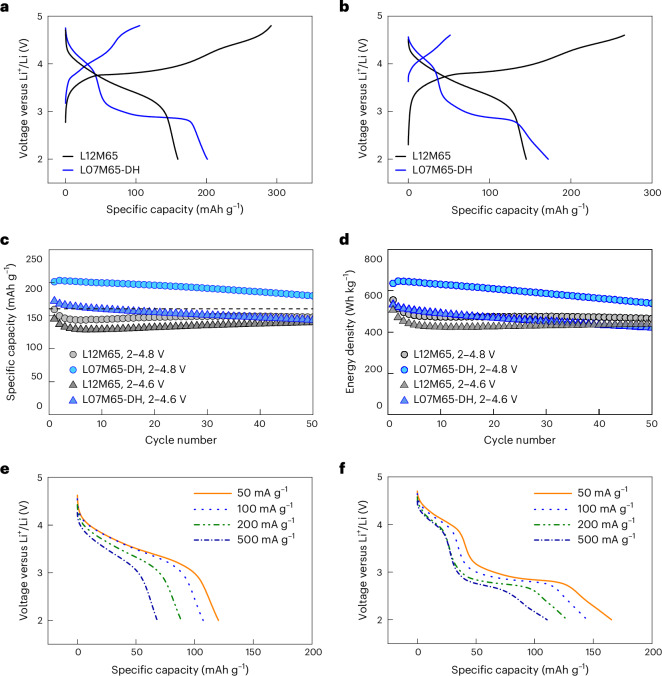
Electrochemical performance of L12M65 and L07M65-DH. **a**,**b**, Voltage profiles of L12M65 and L07M65-DH for the first cycle when cycled between 2 V and 4.8 V (**a**) and 2 V and 4.6 V (**b**) at 20 mAh g^−1^. **c**, Specific capacity retention of L12M65 and L07M65-DH when cycled in different voltage windows at 20 mA g^−1^. **d**, Specific energy retention of L12M65 and L07M65-DH when cycled in different voltage windows at 20 mA g^−1^. **e**,**f**, Rate performance of L12M65 (**e**) and L07M65-DH (**f**) electrodes at the first cycle discharge measured between 2 V and 4.8 V at 50, 100, 200 and 500 mA g^−1^. Source data

The electrochemical performance of L07M65-DH and L12M65 was evaluated using galvanostatic cycling between 2 V and 4.8 V at 20 mAh g^−1^. Both samples retained a large average particle size of ∼5 μm after fabrication into a cathode film (Supplementary Fig. 1d–f). As shown in Fig. 5a, L07M65-DH delivers 201 mAh g^−1^ in the first discharge, substantially higher than the 159 mAh g^−1^ for L12M65. This improvement is maintained in the narrower voltage window of 2–4.6 V (Fig. 5c). Even higher performance of 240 mAh g^−1^ can be achieved by milling down the particle size (Supplementary Fig. 11). Whereas L12M65 (black) shows a sloping voltage profile, L07M65-DH (blue) shows plateau-like 4 V and 3 V regions, with the 3 V region longer than the 4 V region. L07M65-DH achieves a higher maximum specific energy in Fig. 5d (645 Wh kg^−1^ between 2 V and 4.8 V versus 555 Wh kg^−1^ for L12M65). We compare the performance of L07M65-DH to that of ordered spinel in Supplementary Fig. 12 and show that it delivers higher capacity retention. The hard X-ray spectroscopy and soft X-ray mapping of the resonant inelastic X-ray scattering (mRIXS) reveal that both manganese and oxygen redox contribute to the high capacity (Supplementary Figs. 13–15 and Supplementary Notes 2 and 3). We found that the capacity increase from L12M65 to L07M65-DH is accompanied by improvements in rate capability. We tested the performance of L12M65 and L07M65-DH at current densities ranging from 50 mA g^−1^ to 500 mA g^−1^. While the pristine L12M65 only delivers 68 mAh g^−1^ at 500 mA g^−^1 (43% of that at 20 mA g^−1^) (Fig. 5e), modification into L07M65-DH improves capacity at 500 mA/g to 110 mAh g^−1^ (Fig. 5f). Such high specific energy and rate capability have previously only been obtained in nanosized DRX materials but never in micrometre-sized particles.

**Fig. 6:**
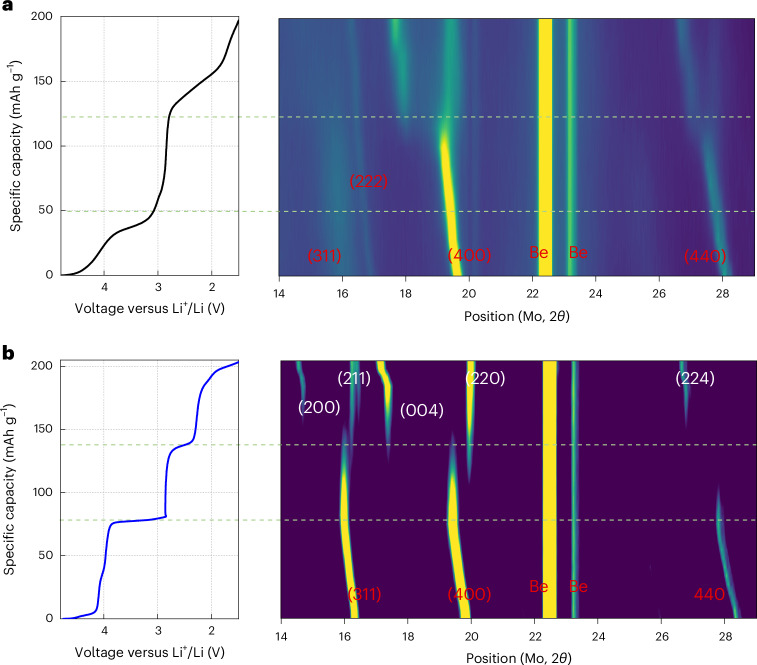
In situ XRD for L07M65-DH and LiMn_2_O_4_ during the first cycle. **a**,**b**, Voltage profile (left) and in situ XRD pattern (right) of L07M65-DH (**a**) and LiMn_2_O_4_ (**b**). The current rate is 20 mA g^−1^. The X-ray scan was performed every 30 min. Cubic spinel peaks are marked in red, and tetragonal spinel peaks are marked in white. Source data

To understand the structural processes upon electrochemical cycling, we show in Fig. 6a in operando diffraction data obtained during the cycling of L07M65-DH between 1.5 V and 4.8 V at 20 mA g^−1^. For comparison, data for the cycling of well-ordered LiMn_2_O_4_ spinel under the same conditions are shown in Fig. 6b. For L07M65-DH the (222), (400) and (440) peaks continuously shift to lower angles upon lithium insertion, consistent with a continuous increase in lattice parameter. When discharged to below 2.7 V, the (400) and (440) peaks split, indicating the formation of the tetragonal phase. However, the lattice parameter for the cubic and tetragonal phases continuously change during discharge, indicating that the system remains a solid solution at all times. In contrast, the data for LiMn_2_O_4_ in Fig. 6b show that the (311), (400) and (440) peaks remain at the same angle when discharging across the 3 V plateau region while their intensity gradually decreases.

## Nanoscale structural effects on electrochemical performance

The attractive properties of manganese make it an important transition metal to enable the scaling of lithium-ion batteries to multiple TWh yr^−1^ production. DRX cathodes can incorporate manganese as an active redox couple but often have limited rate capability unless nanosized^15,16^. In this work we show a unique nanostructured, but large-particle-sized, manganese-based cathode material with high specific energy and capacity, and good rate capability. Although the voltage curves of this δ-phase are reminiscent of well-ordered LiMn_2_O_4_ spinel, significant differences exist between the two: unlike regular spinel, the δ-phase shows no two-phase reaction near 3 V and lithiates as a solid solution in this region^26,28^. In addition, the capacity in the 3 V region is larger than in the 4 V region. The distinct lithiation behaviour between the δ-phase and regular spinel is important because the inhomogeneity resulting from the two-phase reaction near 3 V in the latter leads to particle cracking and degradation of the cathode material. Although recent work has shown that the two-phase reaction can be removed by creating cation disorder through mechanochemical synthesis, the milling process is not practical for scale-up^34–36,16^. The δ-phase material also has better capacity and rate capability than untransformed DRX of similar particle size, similar to the δ-phase-like transformation observed when high-manganese-content DRX materials are transformed slowly into a new structure by electrochemical cycling^26,28^.

In this work, we identify the unique nanoscale microstructure in the δ-phase that is responsible for its distinct electrochemical behaviour. We also show that the δ-phase can be formed through an ex situ process of chemical delithiation and low-temperature heat treatment, which significantly decreases the time required for the DRX-to-δ transformation from 3 weeks (with electrochemical cycling) to 2 days, with an average particle size of ∼10 μm (Supplementary Fig. 1c).

The XRD and SEND analyses present a detailed characterization of the L12M65 sample as it transforms towards δ. Delithiation and heating reveal cell doubling peaks, characteristic of spinel-type cation ordering in L07M65-DH, with a (111) peak at 2.13° and a (311) peak at 4.21° as shown in Fig. 1d. In parallel, SEND analysis (Fig. 3c and Supplementary Fig. 3) indicates that δ-phase evolution, which had started on the surface of the particle immediately after chemical delithiation of L12M65 (Fig. 3f), proceeds to a uniform transformation throughout the particle after mild heating (Fig. 3i). These SEND findings are consistent with the XRD data. L07M65-D displays a weak (111) XRD peak (Fig. 1c), signalling an incomplete transformation to the δ-phase. Upon heat treatment, however, the δ-phase’s characteristic XRD peaks intensify significantly (Fig. 1d) yet retain their broad profile. Even after a complete transformation the broadness of the XRD peaks with odd *l* index persists, consistent with the small domain size indicated by the STEM-HAADF data. This multimodal characterization clearly shows that the δ-phase differs from an ordered spinel and is characterized by cation ordering with a short coherence length, which in turn is critical for the solid-solution behaviour in the 3 V region.

The atomic structure of the δ-phase depicted in the STEM-HAADF image (Fig. 4d) shows partially disordered spinel domains with a coherence length ranging from 3 nm to 7 nm, separated by antiphase boundaries. The antiphase boundaries that separate the spinel domains are thin (<1 nm) and are disordered as shown in Fig. 4d and Supplementary Fig. 10. The presence of antiphase boundaries reflects the high symmetry of the parent DRX phase, and allows for the formation of distinct spinel variants. The process that occurs during delithiation and heating (or cycling^26,28^) therefore seems to be one where spinel ordering starts in DRX in a randomly selected domain variant, which then grows until it meets another variant, separated by an antiphase boundary. This understanding highlights the unique relationship between the δ-phase and DRX. Only when spinel forms from a DRX rocksalt structure does it create a large number of spinel variants which can remain nanosized by impinging on each other at antiphase boundaries. The size of these domains appears remarkably consistent and stable at ∼3–7 nm (ref. ^26^), indicating that their further growth is limited, potentially by a lack of driving force or by the presence of the immobile titanium ions which cannot easily move between octahedral sites.

The nanoscale domain structure and the small coherence length of partially disordered spinel-like domains are key to the electrochemical performance of the δ-phase. These properties effectively suppress the two-phase reaction, similar to what has been observed in nanomaterials^37^. Reducing coherence length in a material is known to transform first-order transformations into second-order ones, or to completely remove them^36,38–40^. In the δ-phase, the small coherence length has remarkable effects on the electrochemical performance, removing the 3 V plateau and turning the associated phase transition into a solid solution region (Fig. 6a). This key feature of the δ-phase eliminates any phase transformation strain as a potential degradation mechanism of the cathode material and enables cycling of a manganese-based spinel over its full theoretical capacity range.

The δ-phase may be an ideal intermediate between fully disordered DRX compounds and well-ordered spinels. In DRX materials, the randomly distributed cations create a wide lithium-site energy distribution, which leads to a sloping voltage profile and reduced lithium diffusivity, necessitating a small particle size in the cathode^41,42^. By transforming the DRX material into a more ordered partial spinel-type structure with small coherence length, the energy density increases due to the increase in capacity delivered in the 4 V and 3 V regions^26^. The formation of the δ-phase also improves rate performance, consistent with previous theoretical work showing that spinel-like configurations possess a more extended low-barrier 0-TM percolation network that is favourable for lithium transport^30,35^. In the δ-phase, more of the capacity is delivered at a higher voltage, which is beneficial for power delivery in practical batteries. The δ-phase approaches spinel-like properties in terms of voltage profile and rate, while retaining a small-enough coherence length to remain as a solid solution and preventing the two-phase reaction in an ordered spinel (Fig. 6).

## Conclusion

Due to the unusual nanoscale features of its microstructure, the δ-phase L07M65-DH delivers a capacity of 201 mAh g^−1^ and high rate capability (Fig. 5) with an average particle size of 4.7 μm (Supplementary Fig. 1f). In contrast, typical DRX materials can only deliver high capacity and energy density with a particle size of <500 nm (ref. ^15^). For example, recent work reported that DRX Li_1.3_Mn_0.4_Nb_0.3_O_2_ delivers only 101 mAh g^−1^ discharge capacity when the average particle size is at the micrometre level^43^. By creating δ-phase material in which the particle size is decoupled from the spinel domain size, a maximum specific energy of 645 Wh kg^−1^ could be achieved in L07M65-DH, which is higher than commercial cathode materials such as LiMn_2_O_4_ and LiFePO_4_, and approaching the values reported for LiNi_1/3_Co_1/3_Mn_1/3_O_2_ (refs. ^44–48^). The crystal density of the δ-phase is ∼4 g cm^−3^, placing it between LiFePO_4_ and NMC cathode materials. Our results show the benefits of engineering local order and microstructure at the nanoscale and show a promising direction for Earth-abundant manganese-based cathode materials.

## Methods

### Synthesis

Li_1.2_Mn_0.65_Ti_0.15_O_1.9_F_0.1_ (L12M65) and LiMn_2_O_4_ were synthesized by a solid-state method. For synthesizing L12M65, Li_2_CO_3_ (Sigma, 99%), Mn_2_O_3_ (Alfa Aesar, 99%), TiO_2_ (Alfa Aesar, 99.6%), MnO_2_ (Alfa Aesar, 99.9%) and LiF (Alfa Aesar, 99.99%) were used as precursors. For LiMn_2_O_4_, Li_2_CO_3_ (Sigma, 99%) and MnO_2_ (Alfa Aesar, 99.9%) were used as precursors. A total amount of 3 g of precursors were mixed in ethanol with a Retsch PM 200 planetary ball mill at a rate of 250 rpm for 12 h. An excess of 10% Li_2_CO_3_ for L12M65 and 5% Li_2_CO_3_ for LiMn_2_O_4_ was added to compensate for possible loss during synthesis. The mixed precursors were then dried at 70 °C overnight before being ground and pelletized. For L12M65, the pellet was sintered at 1,100 °C for 20 min under argon gas flow. For LiMn_2_O_4_, the pellet was sintered at 800 °C for 16 h in air. Li_0.7_Mn_0.65_Ti_0.15_O_1.9_F_0.1_ (L07M65-D) was obtained by adding 400 mg L12M65 into 24 ml 0.1 M NO_2_BF_4_ acetonitrile solution and reacting at 45 °C for 2 days. The solution was washed with acetonitrile and centrifuged at 7,450*g* for 5 min, four times. The sample was dried overnight under vacuum. L07M65-DH was synthesized by pelletizing L07M65-D and heating at 200 °C for 2 h in a sealed quartz tube under vacuum and quenching to room temperature.

### Electrochemistry

To make the cathode film, 70 mg of the as-synthesized active materials and 20 mg Super C65 carbon black (Timcal) were mixed with a mortar and pestle by hand for 30 min. The composite powder was then mixed with PTFE (Dupont) in a 9:1 ratio. The mixed composite was rolled into free-standing thin films and cut with a 5/16th-inch-diameter punch. The electrolyte used is 1 M LiPF_6_ in ethylene carbonate and dimethyl carbonate solution (1:1 v/v, Sigma-Aldrich). Glass microfibres (Whatman) were cut with a 16 mm punch and used as separators. Lithium (MSE Supplies) round metal foil (7/16th inches in diameter) was used as the anode. After assembling the coin cells (CR2032), they were allowed to rest for 4 h before being tested on an Arbin battery cycler at 25 °C. All cathode film fabrication and coin cell assembly procedures were performed in an argon-filled glovebox. The rate capability test was conducted by charging and discharging at the same rate (that is, 50, 100, 200 and 500 mA g^−1^) between 2 V and 4.8 V. A 1 min rest was implemented at the top of the charge and at the bottom of the discharge.

### Characterization

Synchrotron XRD patterns were collected at Beamline 28-ID-2 of the National Synchrotron Light Source II, Brookhaven National Laboratory. All the synchrotron refinements were conducted using the TOPAS software package. A single-phase rocksalt structure model was used when refining the XRD patterns of L12M65 and L07M65-D. For L07M65-DH, a single-phase spinel structure model was used. The reflections with an odd *l* index, which are generated by spinel-type cation ordering, show broader peaks than those with an even *l* index, which are generated by the face-centred cubic cation and anion framework. Therefore, we applied selective peak broadening to capture the broad peaks with an odd *l* index, as applied in a previous publication on the δ-phase^31^. The broadening factor applied to the group of selected peaks is directly related to the size of spinel-like ordering, yielding an estimated coherence length for such environment. SEM images were collected using a Zeiss Gemini Ultra-55 analytical field emission SEM at the Molecular Foundry in Lawrence Berkeley National Laboratory. In situ XRD was performed on a Bruker D8 Advanced X-ray diffractometer using Mo Kα radiation. An in situ cell with a beryllium window was galvanostatically cycled at 20 mA g^−1^ between 1.5 V and 4.8 V using a Maccor potentiostat, and XRD patterns were collected from 14° to 29° 2*θ* every 30 min at room temperature.

### Differential electrochemical mass spectrometry

Outgassing of the Li_1.2_Mn_0_._65_Ti_0.15_O_1.9_F_0.1_ (L12M65) and Li_0.7_Mn_0_._65_Ti_0.1_O_1.9_F_0.1_ (L07M65-DH) cathodes during the first cycle of galvanostatic charge and discharge was monitored on a custom-built differential electrochemical mass spectrometry (DEMS) system, which was operated as described in a previous study^49^. Modified Swagelok-type cells were prepared in an argon-filled glovebox. The compositions of the cathodes and anodes were identical to those of the coin cells used for electrochemical testing in this study. For each DEMS cell, one sheet of Celgard 2500 (polypropylene) and one sheet of QM-A quartz microfibre filters (Whatman) were used as separators with 80 ml of 1 M LiPF_6_ (Gotion) in ethylene carbonate/diethyl carbonate (BASF, 1:1 v/v) added as the electrolyte. The DEMS cells were cycled at a constant current rate of 20 mA g^−1^ under a static head of argon pressure (∼1.2 bar) at room temperature. Any accumulated gas in the cell was purged by 500 ml of argon pulsed every 10 min. The swept-out gas was sent to a holding chamber, from where it was subsequently leaked into a mass spectrometry chamber for analysis. The set-up was calibrated for O_2_ and CO_2_ in the carrier gas argon.

### SEND

L12M65, L07M65-D and L07M65-DH cathode particles were dispensed onto TEM lacey carbon grids with formvar support inside an argon-filled glovebox, without being exposed to any solutions during the TEM sample preparation. The SEND patterns were collected using a Titan 80-300 scanning/transmission electron microscope operated at 300 kV with a Gatan K3 direct electron camera operating in counting mode. The indicated semiconvergence angle was 0.62 mrad, and the diffraction patterns were energy-filtered with a 15 eV slit centred around the zero-loss peak. The SEND patterns were analysed using the py4DSTEM v.0.14.8 package^50^.

To reconstruct a virtual image of the spinel phase of the L07M65-D sample, only peaks unique to the spinel phase were integrated. These peaks are marked as green dashed boxes in Supplementary Fig. 7a. The scattering angles for these peaks are 0.175–0.250 Å^−1^, 0.51–0.54 Å^−1^, 0.61–0.65 Å^−1^, 0.7–0.75 Å^−1^ and 1.09–1.16 Å^−1^. The integrated virtual image was normalized for thickness by integrating the region between the scattering angles 0.175 Å^−1^ and 1.2 Å^−1^.

Similarly, for the L07M65-DH sample the unique spinel phases were integrated as shown by the dashed boxes in Supplementary Fig. 9a. The scattering angles for these peaks are 0.175–0.250 Å^−1^, 0.37–0.41 Å^−1^, 0.52 0.56 Å^−1^, 0.62–0.64 Å^−1^, 0.687 0.74 Å^−1^, 0.845–0.905–Å^−1^, 0.97–1.03 Å^−1^and 1.085–1.17 Å^−1^. The integrated virtual image was normalized for thickness by dividing the spinel virtual image with the virtual image formed by integrating the scattering angle from 0.175 Å^−1^ to 1.2 Å^−1^.

### Atomic-resolution imaging

To prepare the ex situ samples for atomic-resolution TEM imaging, the L07M65-DH cathode particles were mixed with PTFE with a mortar and pestle in a ratio of 9:1. The composite was then rolled into cathode thin film. A thin lamella suitable for TEM analysis was meticulously crafted from the specimen film using the dual-beam focused ion beam method, utilizing a Helios G4 UX instrument. The lamella was thinned down to ∼1 μm using a gallium-ion beam of 30 kV accelerating voltage and 0.75 nA current. To prevent ion beam damage, subsequent thinning to ∼100 nm was performed using a 5 kV accelerating voltage and 0.15 nA current. To remove the amorphous layer and further thin, a low-energy 2 kV beam was used on the nanomill. STEM-HAADF imaging was performed on an aberration-corrected Titan 80-300 scanning/transmission electron microscope operated at 300 kV. The convergence angle was 30 mrad. An adaptive thresholding of 51 pixels and a third-order low-pass Butterworth filter with a frequency cut-off of 160 pixels was applied in the frequency domain to correct for the non-uniform illumination and to filter the high-frequency noise in the HAADF-STEM micrographs. The raw HAADF-STEM micrograph is shown in Supplementary Fig. 10a.

### EELS

The electron energy loss measurements were performed using the TEAM 1 microscope (double-aberration-corrected Thermo Fisher Scientific Titan 80-300) at the National Center for Electron Microscopy. The EELS dataset was collected using a Gatan Continuum spectrometer at 300 kV, using a convergence angle of approximately 30 mrad and a collection angle of 110 mrad. The width of the zero-loss peak was measured to be 0.9 eV, and a 0.18 eV per channel dispersion was used to collect the spectra. The lithium distribution was mapped by analysing the Li K-edge (~55 eV) using Gatan Digital Micrograph v.3.6 software.

### Bragg filtering for atomic-resolution images

Bragg filtering is done by filtering the two spinel peaks, as shown in the inset of Fig. 4c. The filtering of the spinel peaks is based on Fourier-transform-based analysis. The image is first windowed using a Tuckey (or tapered cosine) window. The window considers the size of the window and the fraction of the window that is tapered. It effectively combines a rectangular and cosine window, providing a balance between spatial and frequency resolution. The application of this window function to the image prior to fast Fourier transform significantly reduces spectral leakage, enhancing the accuracy of the subsequent frequency-based analysis. Following the windowing, the image undergoes fast Fourier transform to shift the focus from the spatial domain to the frequency domain. The function then employs filtering masks derived from predefined spinel peak coordinates to isolate the specific frequency components pertinent to the spinel phases. These filtered components are transformed back into the spatial domain to reconstruct images representing the parent and superlattice signals. Only half of the spinel peaks are considered for filtering because the conjugate points contain the same information in a Fourier transform of a real-valued image through the property of Hermitian symmetry^51^. The visualization aspect of the spinel lattice is augmented by calculating the ratio of spinel to parent (DRX) lattice signals. The RGB images in Fig. 4d represent the results. These mathematical operations were achieved using an in-house MATLAB script.

### Ex situ Mn K-edge X-ray absorption spectroscopy

Ex situ X-ray absorption spectroscopy measurements at the Mn K-edge were performed in transmission mode at Beamline 7-BM of the National Synchrotron Light Source II, Brookhaven National Laboratory. A Si(111) monochromator was used to select the incident beam energy. A rhodium-coated mirror was applied to obtain harmonic rejection. All electrode films were made in the same way as described above (Electrochemistry). The electrode films were cycled in coin cells to different states of charge and held at specified voltages for 6 h to reach equilibrium. The cells were disassembled, and the films were washed with diethyl carbonate in an argon-filled glovebox. Energy calibration was accomplished by simultaneously measuring the spectra of manganese metal foil. Normalization and calibration of raw data were performed with Athena software^52^.

### Mapping of resonant inelastic X-ray scattering

Mapping of resonant inelastic X-ray scattering of the O K-edge and the Mn L-edge was performed in the iRIXS end station at Beamline 8.0.1.1 of the Advanced Light Source^53^ at Lawrence Berkeley National Laboratory. The beam spot size is about 25 × 100 mm^2^. Mapping data were collected by an ultrahigh-efficiency modular spectrometer^54^, with an excitation energy step of 0.2 eV. The resolution of the excitation energy is ∼0.2 eV, and that of the emission energy is ∼0.3 eV. The ex situ samples were cathode films, composed of active materials, carbon black and PTFE in a weight ratio of 7:2:1. The cathode films were charged or discharged to a specified state of charge in a coin cell at 20 mA g^−1^ and held at that voltage for 6 h. The cells were disassembled, and the films were washed with diethyl carbonate in an argon-filled glovebox before being transferred to the experimental chamber through a home-made air-free transfer kit. Final two-dimensional maps were achieved via multistep data processing, including normalization of beam flux and collecting time, integration and combination, etc., which has been elaborated on in a previous work^40^.

## Online content

Any methods, additional references, Nature Portfolio reporting summaries, source data, extended data, supplementary information, acknowledgements, peer review information; details of author contributions and competing interests; and statements of data and code availability are available at 10.1038/s41565-024-01787-y.

## Supplementary information

Supplementary Information Supplementary Notes 1–3, Tables 1–4 and Figs. 1–15.

## Source data

Source Data Fig. 1 XRD data in Fig. 1.

Source Data Fig. 2 In situ XRD data in Fig. 2.

Source Data Fig. 5 Electrochemistry data in Fig. 5.

Source Data Fig. 6 Electrochemistry data in Fig. 6.

## Data Availability

All data generated and analysed during this study are included in the published article and its Supplementary Information. Source data are provided with this paper.
