# Supplementary material for: Earth-abundant Li-ion cathode materials with nanoengineered microstructures
Source: Nat Nanotechnol. 2024 Sep 19;19(12):1831–9. doi: 10.1038/s41565-024-01787-y (PMC11638071; doi:10.1038/s41565-024-01787-y)
Supplement: Supplementary file 1 — Supplementary Notes 1–3, Tables 1–4 and Figs. 1–15. [file 41565_2024_1787_MOESM1_ESM.pdf]

nature nanotechnology

Supplementary information

https://doi.org/10.1038/s41565-024-01787-y

Earth-abundant Li-ion cathode materials with nanoengineered microstructures

In the format provided by the authors and unedited

Table of Contents

Supplementary Note 1: Effect of thickness in virtual imaging 2

Supplementary Note 2: Redox mechanism 2

Supplementary Note 3: Oxygen redox characterization in L07M65-DH 3

Supplementary Table 1. Structural information of L12M65 based on synchrotron powder diffraction refinement in Fm-3m space group 4

Supplementary Table 2. Structural information of L07M65-D based on synchrotron powder diffraction refinement in Fm-3m space group 4

Supplementary Table 3. Structural information of L07M65-DH based on synchrotron powder diffraction refinement in Fd-3m space group 4

Supplementary Table 4. Target versus measured atomic ratios of the synthesized material....5

Supplementary Figure 1. Particle analysis of L12M65 and L07M65-DH..6

Supplementary Figure 2. Selected electrochemical performance of L07M65-D annealed at 500 °C and 600 °C 6

Supplementary Figure 3. Radial integration and indexing of selected diffraction patterns from the SEND dataset of a L12M65 particle 7

Supplementary Figure 4. Indexing of selected diffraction patters from the SEND dataset of a L07M65-D particle 10

Supplementary Figure 5. Radial integration of the SEND patterns of a L07M65-DH particle 13

Supplementary Figure 6: Effect of normalization of the virtual imaged spinel peaks 14

Supplementary Figure 7. Indexing of selected diffraction patters from the SEND dataset of L07M65-DH particle 15

Supplementary Figure 8. Radial integration of the SEND patterns from L07M65-DH.........18

Supplementary Figure 9. Multimodal characterization of L07M65-DH..19

Supplementary Figure 10. Fourier filtering of the HAADF-STEM micrograph....................20

Supplementary Figure 11. Electrochemical performance of L07M65-DH with smaller particle size 21

Supplementary Figure 12. Electrochemical performance of $LiMn_2O_4$..21

Supplementary Figure 13. The X-ray absorption near edge structure (XANES) spectra of as-synthesized L12M65 and L07M65-DH at different states of charge 22

Supplementary Figure 14. *Ex-situ* O K-edge mRIXS spectra of L07M65-DH 22

Supplementary Figure 15. Gas evolution measurements 23

Supplementary References 24

Supplementary Notes

Supplementary Note 1: Effect of thickness in virtual imaging

To demonstrate the effect of thickness and prove the approach for the thickness correction, we prepared a FIB sample with uniform thickness as shown in **Fig. S6 A**. The FIB lamella was prepared such that a large particle was visible in either side of the lamella and had a uniform thickness. The δ phases were identified by integrating the scattering angle around the spinel peaks, namely (0.19 to 0.22) Å^{-1}, (0.38 to 0.41) Å^{-1}, (0.51 to 0.54) Å^{-1}$_{1}$, (0.61 to 0.64) Å^{-1}, (0.7 to 0.72) Å^{-1}, and (0.82 to 0.85) Å^{-1}. The virtual image obtained by integrating these peaks is shown in panel C for **Fig. S6A**. From this result it would appear that only the smaller particles undergo the DRX to δ transformation. This virtual image was further normalized with the scattering at each point between (0.175 to 1.2) Å^{-1}. This includes the entire scattering region collected by the electron detector excluding the unscattered central beam. This normalized virtual image is plotted in panel D. This clearly shows all the particles have transformed into the δ phase including the large particle with uniform thickness. Therefore, normalizing the virtual image obtained by integrating the spinel peaks with the total scattering is crucial in understanding the spatial extent of the δ phase.

Supplementary Note 2: Redox mechanism

Hard XAS was applied to qualitatively analyze the redox mechanism L12M65 and L07M65-DH. The Mn K-edge XANES spectra of the compounds are shown in **Supplementary Fig. 13**, where they are compared with MnO, Mn_2O_3, and MnO_2 standards. The Mn K-edge XANES spectrum in **Supplementary Fig. 13A** shows that L12M65 utilizes $Mn^{3/4+}$ during cycling, and the utilization of Mn redox increases from cycle 1 to 20 as the material undergoes DRX-to-δ transformation. The results in **Supplementary Fig. 13B** indicate that the Mn valence increases

during chemical delithiation and that the edge position remains constant during heating, confirming that Li is extracted upon delithiation and that heating does not vary the valence state of Mn in L07M65-DH. In **Supplementary Fig. 13C**, L07M65-DH mainly utilizes Mn3/4+ redox during the first 20 cycles when cycling between 1.5-4.8 V. L07M65-DH delivers higher discharge capacity than the theoretical Mn^{3+}/Mn^{4+} capacity available, indicating that oxygen redox is likely to contribute capacity, which may explain the Mn reduction observed for L07M65-DH.[1]

Supplementary Note 3: Oxygen redox characterization in L07M65-DH

The soft X-ray O *K*-edge mapping of the resonant inelastic X-ray scattering (mRIXS) spectrum of L07M65-DH is shown in **Supplementary Fig. 14**. Previous studies have demonstrated that there are two characteristic features, both at 531.0 eV excitation energy, associated with oxidized oxygen states in fully charged electrodes involving oxygen redox reactions: i) a feature at 523.7 eV emission energy, and ii) low energy excitation features close to the elastic peak, i.e., at the same 531.0 eV emission energy as the excitation energy.[2-6] As shown in **Supplementary Fig. 14**, L07M65-DH does not show such features in the pristine state. However, both oxygen redox features appear when charged to 4.8 V at the first cycle. This indicates that oxygen contributes to the redox mechanism in L07M65-DH and explains why L07M65-DH delivers higher than theoretical capacity expected solely from Mn^{+3}/Mn^{+4} redox. In **Supplementary Fig. 15**, we further conducted differential electrochemical mass spectrometry (DEMS) for L12M65 and L07M65-DH, and no oxygen release is detected for both samples. The results indicate that there is limited irreversible oxygen redox in both materials. In addition, CO_2 emission was measured. L12M65 shows higher CO_2 evolution than L07M65-DH. As shown in previous work on Mn-based DRX cathodes, CO2 release can arise from Li_2CO_3 decomposition left from the Li-excess in the synthesis, or from breakdown of the EC solvent at carbon in the electrode.[7]

Supplementary Table 1. Structural information of L12M65 based on synchrotron powder diffraction refinement in Fm-3m space group.

Space group: Fm-3m; a = b = c = 4.134(4); Rwp = 8.56 %						
Atom	Wyckoff symbol	*x*	*y*	*z*	B_{iso}	Occupancy
Li	4a	0	0	0	0.5	0.6
Mn^{+3}/Ti1	4a	0	0	0	0.5	0.25
Mn^{+4}/Ti2	4a	0	0	0	0.5	0.075
Ti	4a	0	0	0	0.5	0.075
O	4b	0.5	0.5	0.5	0.5	0.95
F	4b	0.5	0.5	0.5	0.5	0.05

Supplementary Table 2. Structural information of L07M65-D based on synchrotron powder diffraction refinement in Fm-3m space group.

Space group: Fm-3m; a = b = c = 4.068(0); Rwp = 9.33 %						
Atom	Wyckoff symbol	*x*	*y*	*z*	B_{iso}	Occupancy
Li	4a	0	0	0	0.5	0.35
Mn^{+3}/ Ti1	4a	0	0	0	1.10(8)	0.25
Mn^{+4}/ Ti2	4a	0	0	0	1.10(8)	0.075
Ti	4a	0	0	0	1.10(8)	0.075
O	4b	0.5	0.5	0.5	1.11(2)	0.95
F	4b	0.5	0.5	0.5	1.11(2)	0.05

Supplementary Table 3. Structural information of L07M65-DH based on synchrotron powder diffraction refinement in Fd-3m space group.

Space group: *Fd-3m*; a = b = c = 8.18(4) Å; *Rwp* = 8.86 %						
Atom	Wyckoff symbol	*x*	*y*	*z*	B_{iso}	Occupancy
Li	8a	0.125	0.125	0.125	0.35	0.2
Mn^{+3}/Ti1	16d	0.5	0.5	0.5	0.97(6)	0.73(7)
Mn^{+4}/Ti2	16c	0	0	0	0.97(6)	0.06(3)
O	32e	0.2628	0.2628	0.2628	1.49(1)	0.95
F	32e	0.2628	0.2628	0.2628	1.49(1)	0.05

Supplementary Table 4: Target versus measured atomic ratios of the synthesized materials

	Target atomic ratio (Li: Mn: Ti)	Measured atomic ratio (Li: Mn: Ti)	Measured atomic ratio (Li: Mn: Ti)
$Li_{1.2}Mn_{0.65}Ti_{0.15}O_{1.9}F_{0.1}$ (L12M65)	1.2:0.65:0.15	Based on cation sum=2 1.218:0.653:0.129	Based on cation sum=2 1.218:0.653:0.129
$Li_{0.7}Mn_{0.65}Ti_{0.15}O_{1.9}F_{0.1}$ (L07M65-D)	0.7:0.65:0.15	Normalized with Mn 0.589:0.65:0.158	Normalized with Ti 0.559:0.616:0.15
$Li_{0.7}Mn_{0.65}Ti_{0.15}O_{1.9}F_{0.1}$ (L07M65-DH)	0.7:0.65:0.15	Normalized with Mn 0.544:0.65:0.153	Normalized with Ti 0.533:0.636:0.15

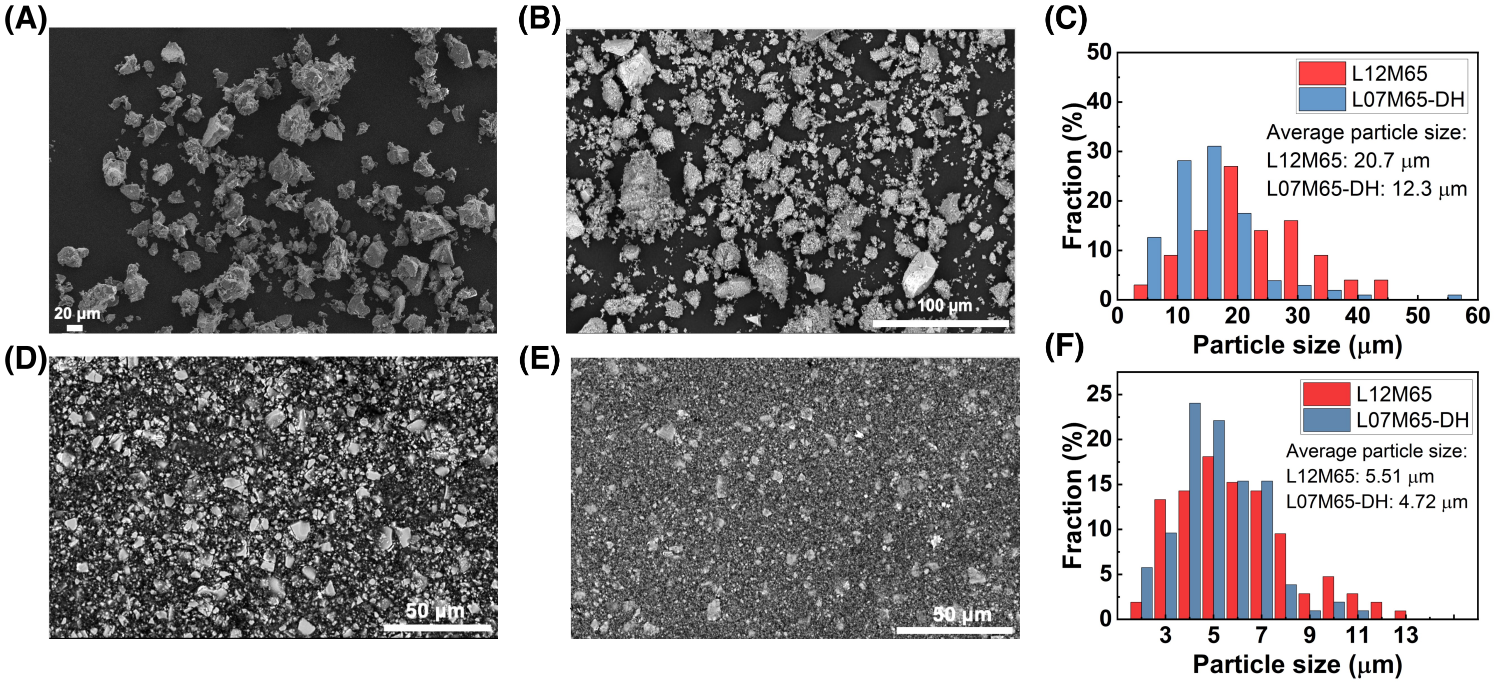

Supplementary Figure 1. Particle analysis of L12M65 and L07M65-DH. (**A** to **B**) The SEM images of as-synthesized **(A)** L12M65 and **(B)** L07M65-DH. **(C)** Statistical analyses of the particle sizes of L12M65 and L07M65-DH obtained from Figures **(A)**-**(B)**. (**C** to **D**) The SEM images of **(C)** L12M65 and **(D)** L07M65-DH prior to electrochemical cycling. **(E)** Statistical analyses of the particle sizes of L12M65 and L07M65-DH obtained from Figures **(D)**-**(E)**.

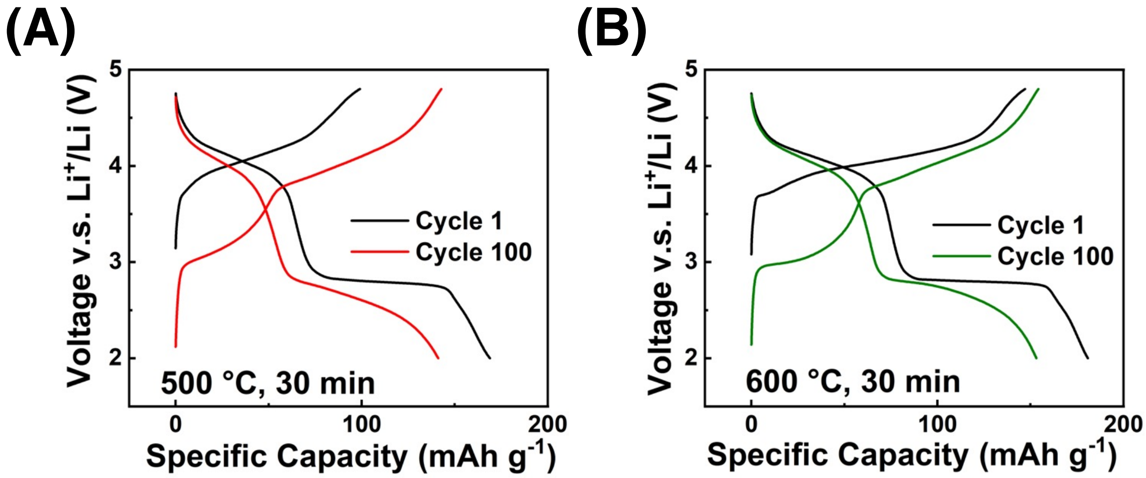

Supplementary Figure 2. Selected electrochemical performance of L07M65-D annealed at 500 °C and 600 °C. Voltage profiles L07M65-D heated at **(A)** 500 °C and **(B)** 600 °C for 30 mins between 2 and 4.8 at 20 mAh/g.

(A)

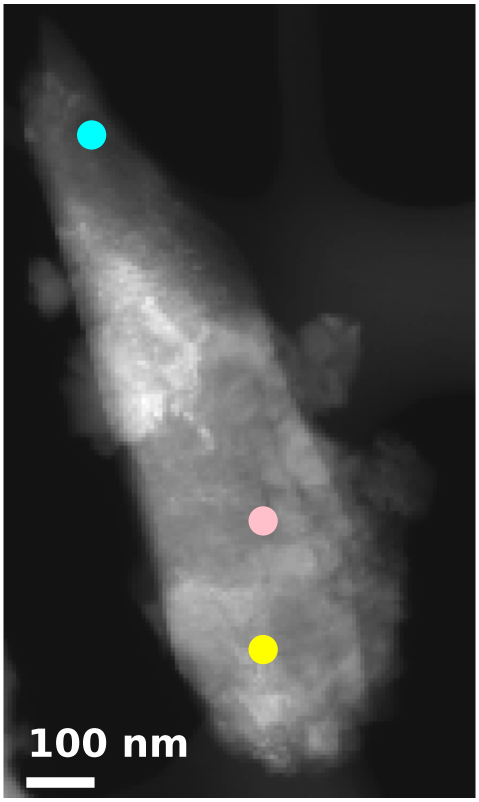

(B)

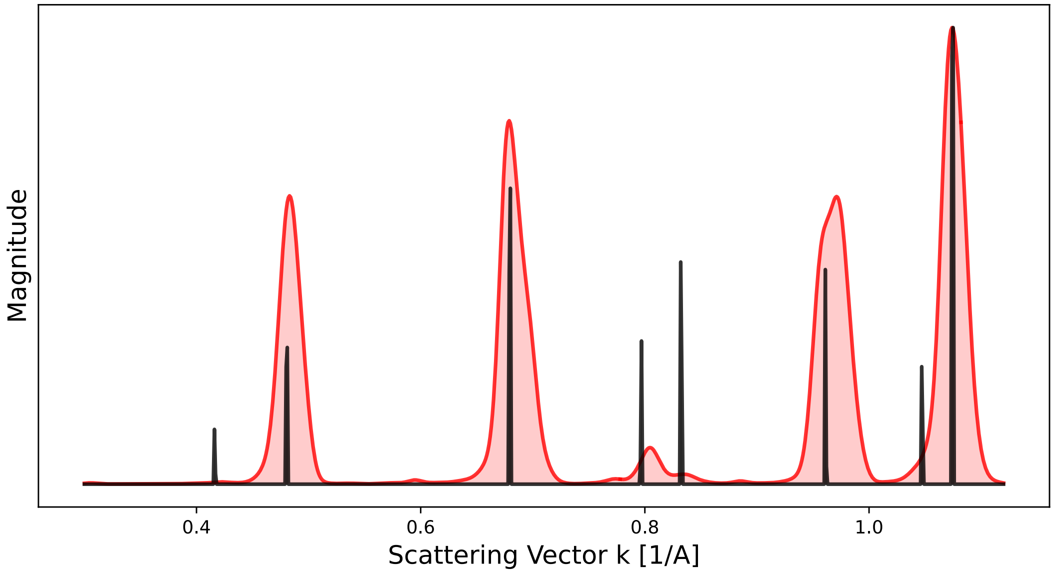

(C)

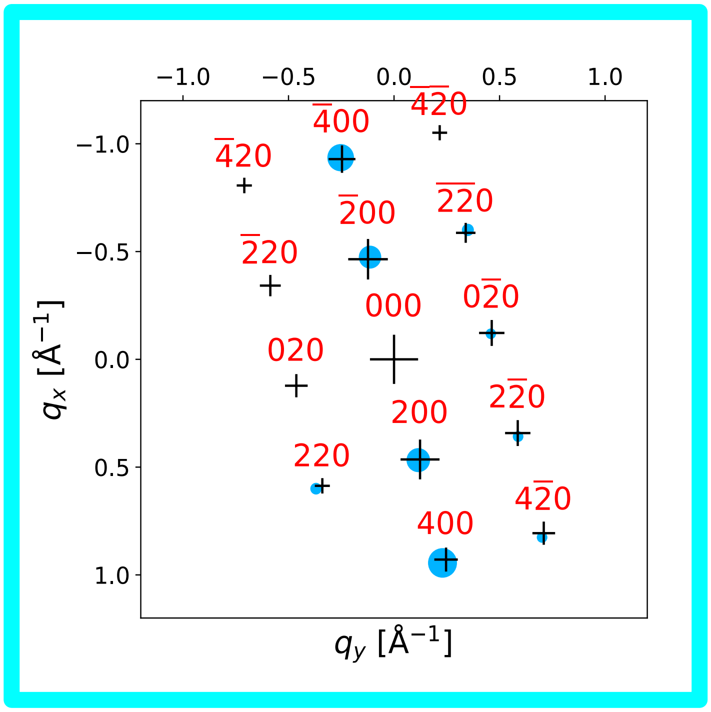

−1.0
−0.5
0.0
0.5
1.0
$\bar{4}\bar{2}0$
$\bar{4}00$
$\bar{4}20$
$\bar{2}00$
$\bar{2}\bar{2}0$
$\bar{2}20$
$0\bar{2}0$
000
020
$2\bar{2}0$
200
220
$4\bar{2}0$
400
q_x [Å^{-1}]
q_y [Å^{-1}]

(D)

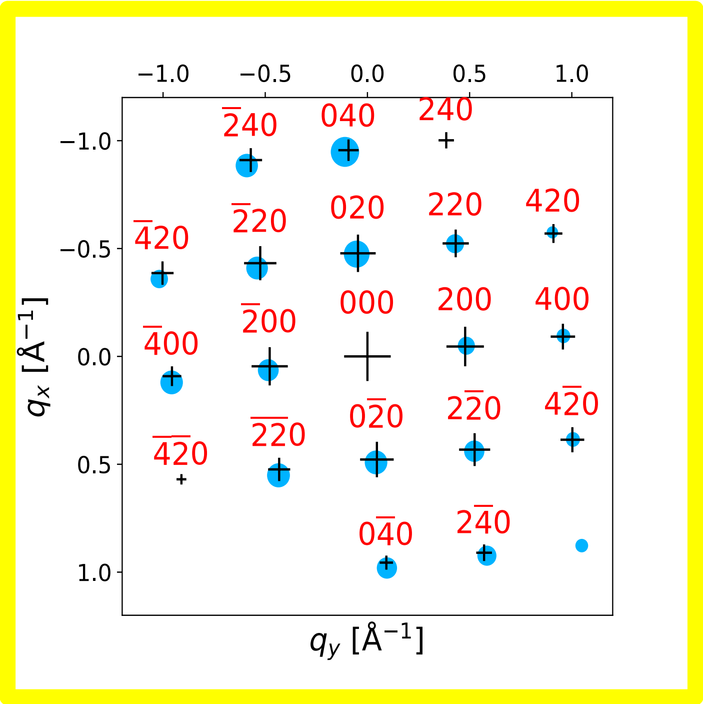

−1.0
−0.5
0.0
0.5
1.0
$\bar{2}40$
040
240
$\bar{4}20$
$\bar{2}20$
020
220
420
$\bar{4}00$
$\bar{2}00$
000
200
400
$\bar{4}\bar{2}0$
$\bar{2}\bar{2}0$
$0\bar{2}0$
$2\bar{2}0$
$4\bar{2}0$
$0\bar{4}0$
$2\bar{4}0$
q_x [Å^{-1}]
q_y [Å^{-1}]

(E)

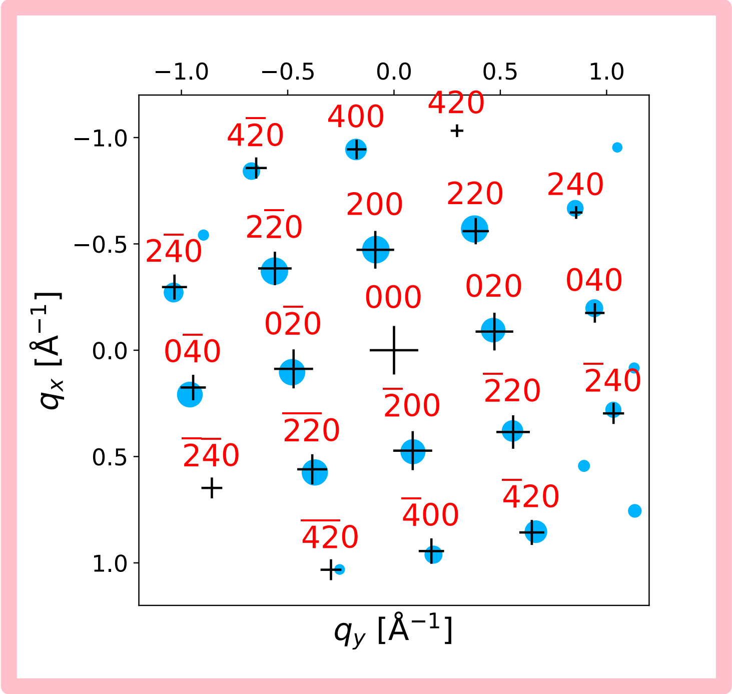

Supplementary Figure 3. Radial integration and indexing of selected diffraction patterns from the SEND dataset of a L12M65 particle. (A) HAADF-STEM micrography of typical L12M65 particle. **(B)** The intensity of SEND patterns radial integrated overlaid with the DRX peaks obtained from the simulation. The intensity of the indexed diffraction pattern from the spots **(C)** cyan, **(D)** yellow, and **(E)** pink marked in the HAADF image. The blue dots represent the experimental diffraction pattern from the point of the same color in the ADF image in panel A. The black crosses represent the simulated diffraction pattern from the structure file obtained from the XRD measurements in the L12M65 samples as given in **Supplementary Table 1**.

(A)

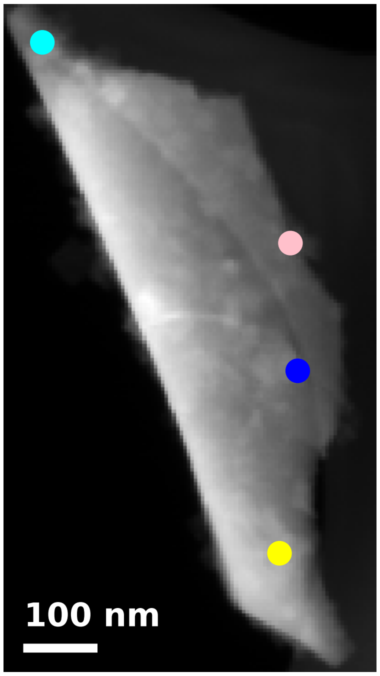

(B)

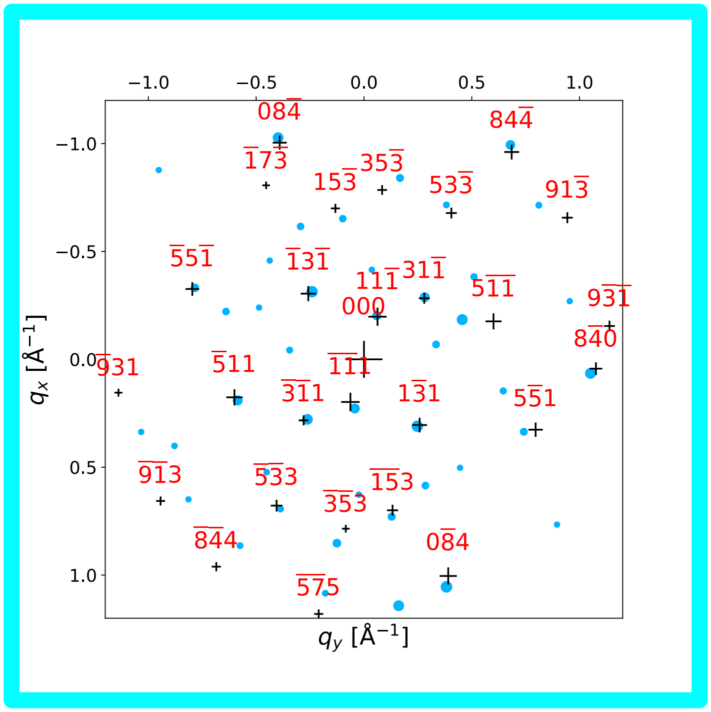

(C)

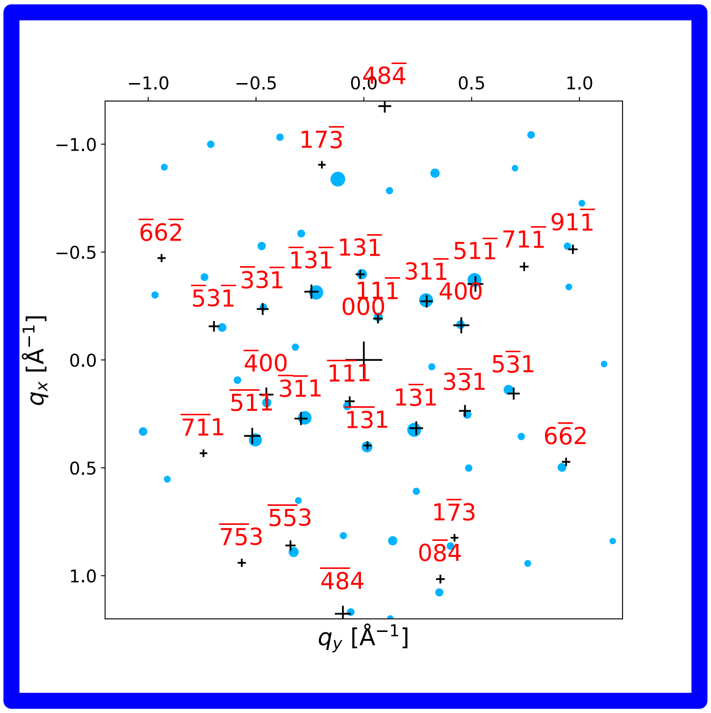

(D)

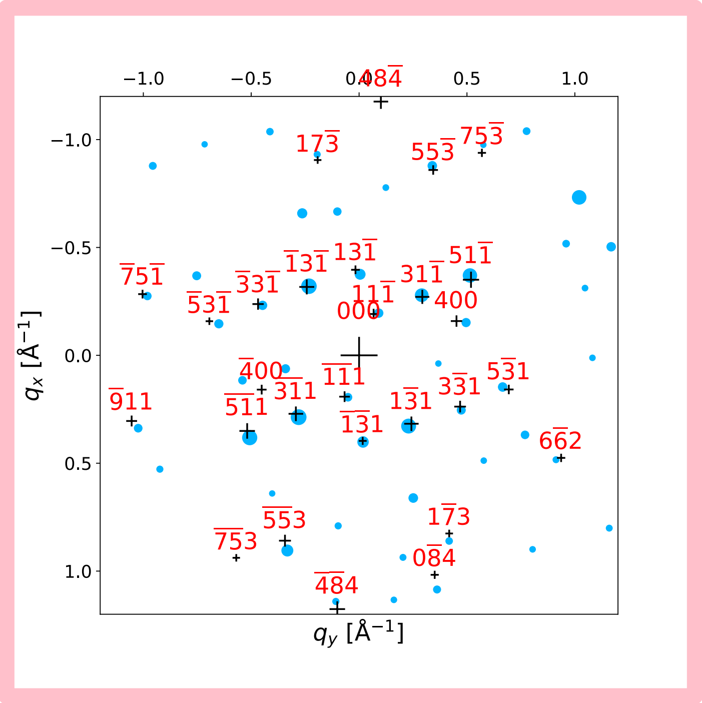

(E)

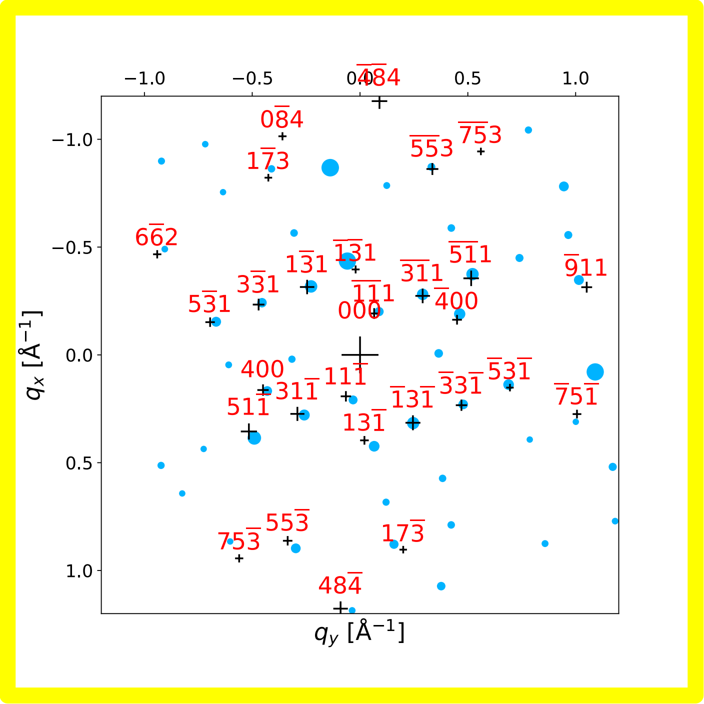

Supplementary Figure 4. Indexing of selected diffraction patters from the SEND dataset of a L07M65-D particle. (A) HAADF-STEM micrography of typical L07M65-D particle. Indexed diffraction pattern from the spots **(B)** cyan, **(C)** blue, **(D)** pink, and **(E)** yellow colors marked in the HAADF image. The blue dots represent the experimental diffraction pattern from the point of the same color in the ADF image on the left. The black crosses represent the simulated diffraction pattern from the structure file obtained from the XRD measurements in the L07M65-DH samples as given in **Supplementary Table 3**.

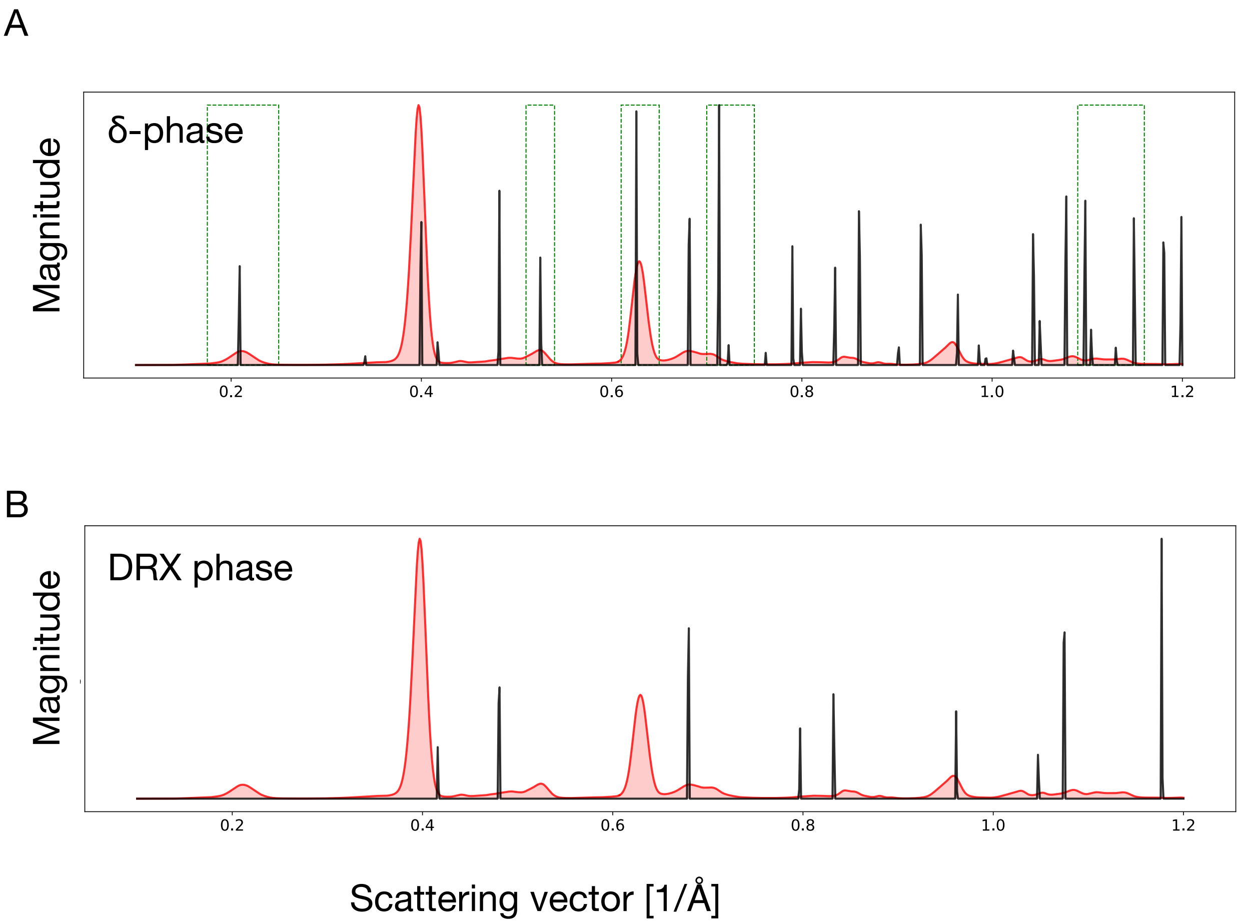

Supplementary Figure 5. Radial integration of the SEND patterns of a L07M65-DH particle. Radial integration of the SEND patterns denoted in red from the L07M65-DH particle shown in Fig. 4D. Panel **(A)** shows the simulated radial profile from the structure file obtained from the XRD of L07M65-DH in black. Panel **(B)** shows the simulated radial profile from the structure file obtained from the XRD of L12M65 (DRX-phase). The unique spinel peaks integrated to obtain the extent of δ-phase in **Fig. 4E** are marked as green dashed boxes in panel A. Crystal planes that are normal to the electron beam will not be represented in the TEM diffraction data which leads to the absence of some of the simulated peaks in the experimental radially integrated diffraction pattern. Additionally, the intensity difference between the experimental and simulated radially integrated patterns would be the result of the kinematical scattering approximation used in the simulation of the powder electron diffraction patterns.

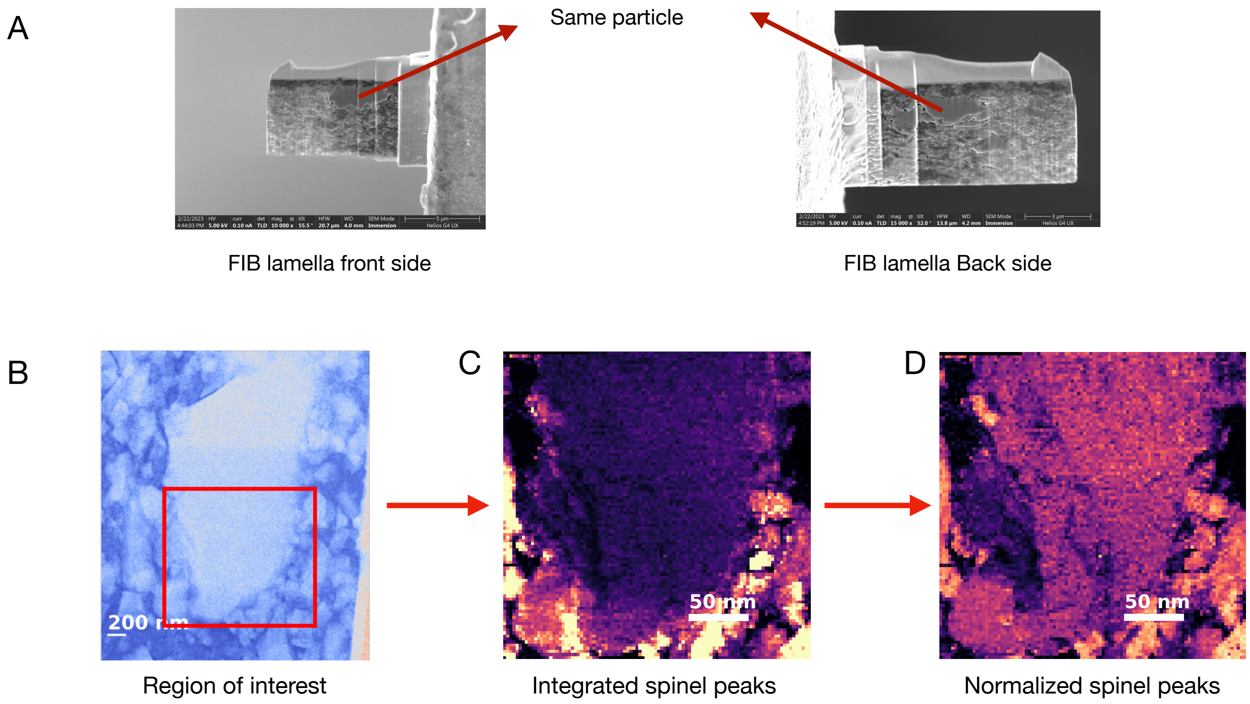

Supplementary Figure 6: Effect of normalization of the virtual imaged spinel peaks. Panel **A** shows a FIB prepared TEM lamella with a large particle with uniform thickness. Panel **B** shows the region of interest where the SEND patterns were collected. The diffraction peaks unique to the spinel peaks were integrated and the virtual image is shown in panel **C**. Panel **D** shows the virtual image after normalizing with the integrated scattering from 0.175 to 1.2 Å^{-1}. The large particle was invisible in the virtual image after integration of the spinel peaks on the account of thickness, whereas smaller particles were bright and visible. Upon normalization all the transformed regions are clearly visible.

(A)

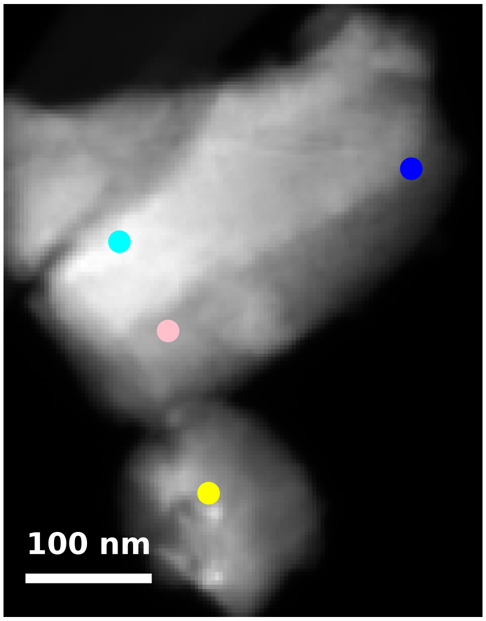

(B)

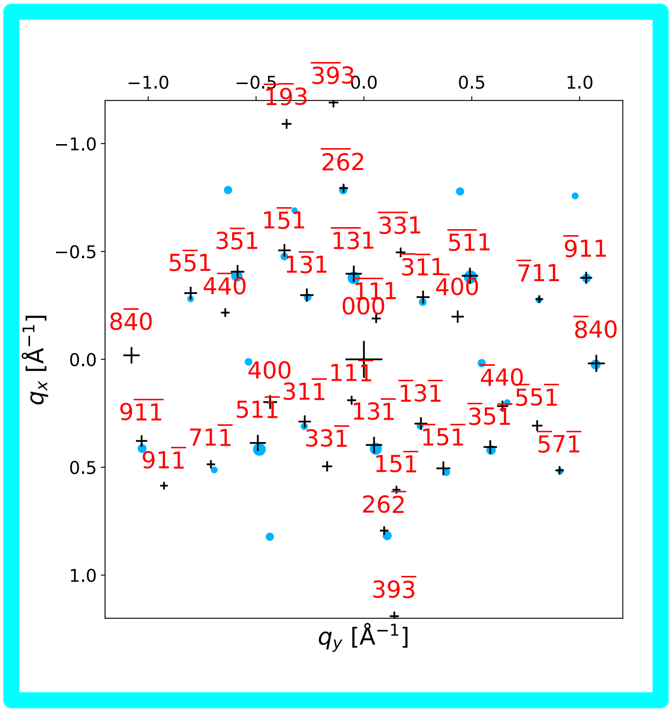

(C)

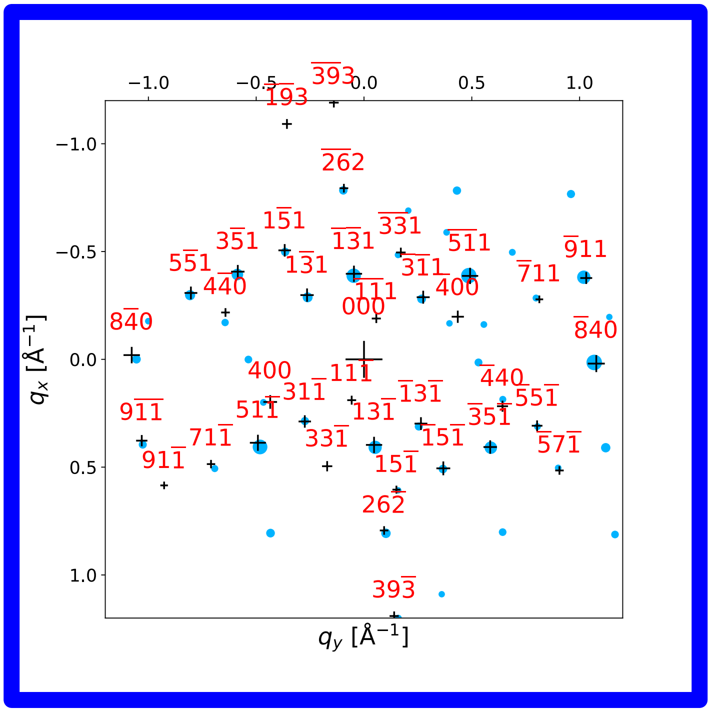

(D)

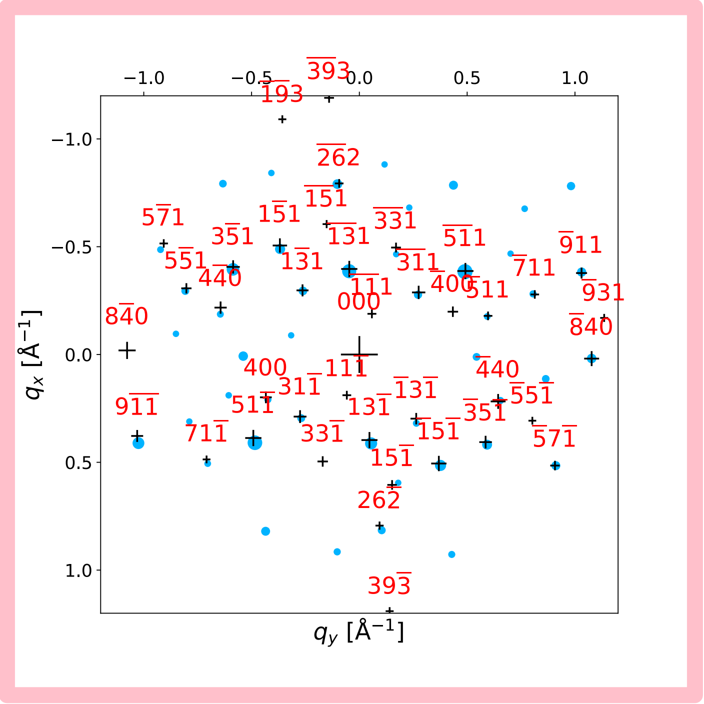

(E)

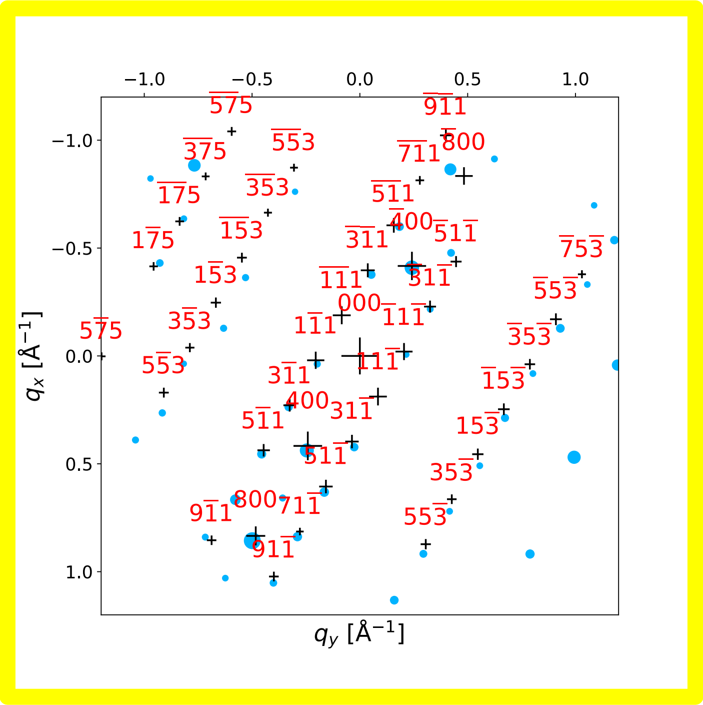

Supplementary Figure 7. Indexing of selected diffraction patters from the SEND dataset of L07M65-DH particle. (A) HAADF-STEM image of a L07M65-DH particle. Indexed diffraction patternes from the spots **(B)** cyan, **(C)** blue, **(D)** pink, and **(E)** yellow marked in the HAADF image. The blue dots represent the experimental diffraction pattern from the point of the same color in the ADF image on the left. The black crosses represent the simulated diffraction pattern from the structure file obtained from the XRD measurements in the L07M65-DH samples as given in **Supplementary Table 3**.

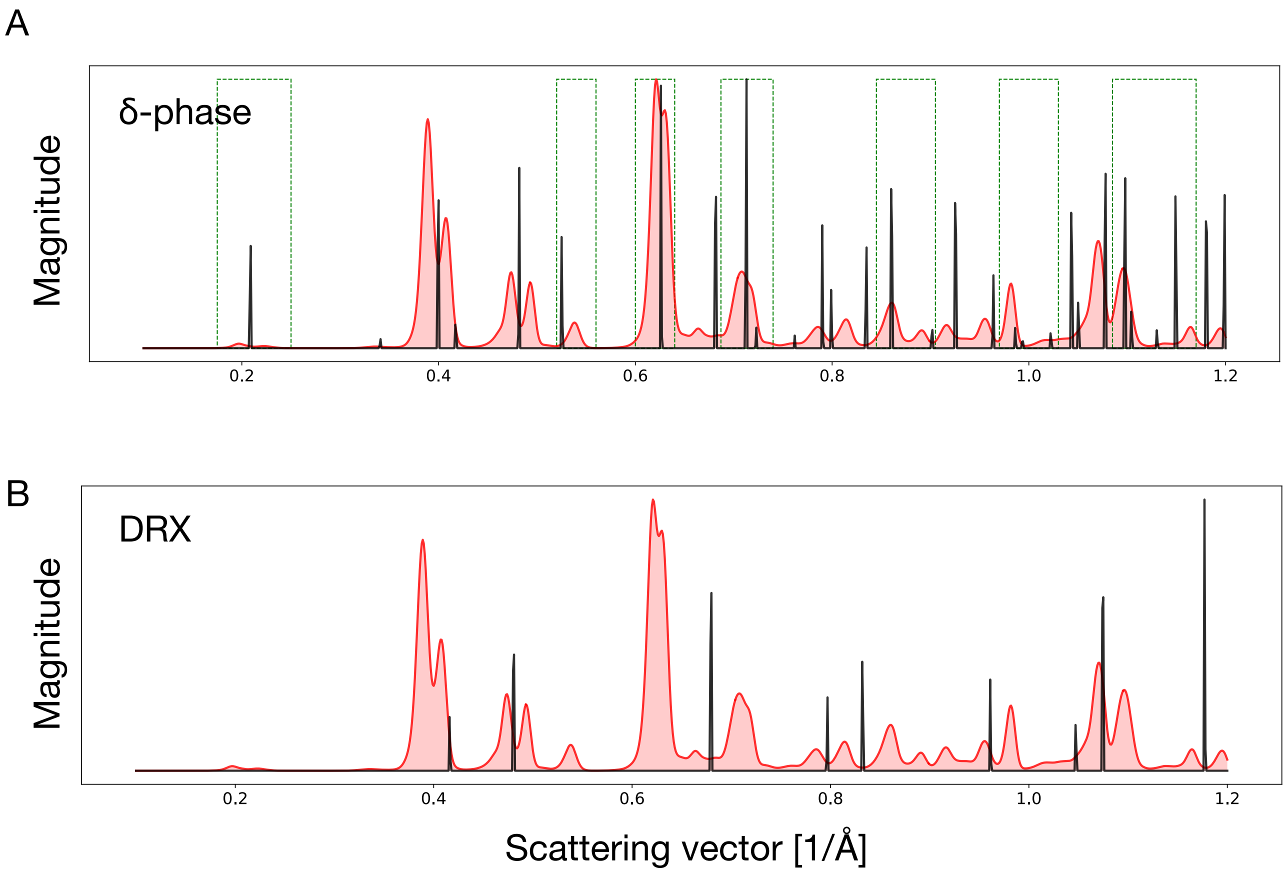

Supplementary Figure 8. Radial integration of the SEND patterns from L07M65-DH. Radial integration of the SEND patterns denoted in red from the L07M65-DH particle shown in Fig. 4G. Panel **(A)** shows the simulated radial profile from the structure file obtained from the XRD of L07M65-DH in black. Panel **(B)** shows the simulated radial profile from the structure file obtained from the XRD of L12M65. The unique spinel peaks integrated to obtain the extent of δ-phase in Fig. 4G are marked as green dashed boxes in panel A. Crystal planes that are normal to the electron beam will not be represented in the TEM diffraction data which leads to the absence of some of the simulated peaks in the experimental radially integrated diffraction pattern. Additionally, the intensity difference between the experimental and simulated radially integrated patterns would be the result of the kinematical scattering approximation used in the simulation of the powder electron diffraction patterns.

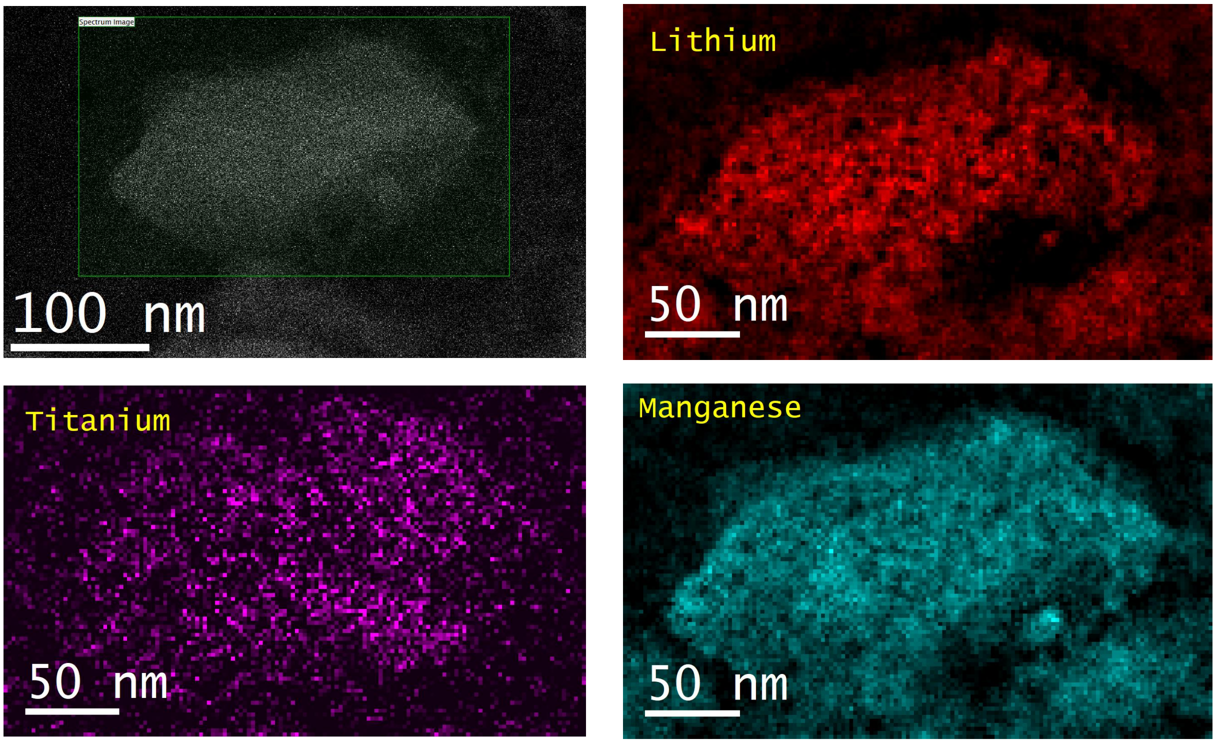

Supplementary Figure 9. Multimodal characterization of L07M65-DH. STEM-HAADF and EELS composition mapping of the same L07M65-DH from which the SEND and atomic resolution HAADF-STEM datasets in Fig. 4 were collected. The Li, Ti, and Mn maps were drawn using the K-edge (55 eV), L-edge (456 eV), and M-edge (49 eV), respectively.

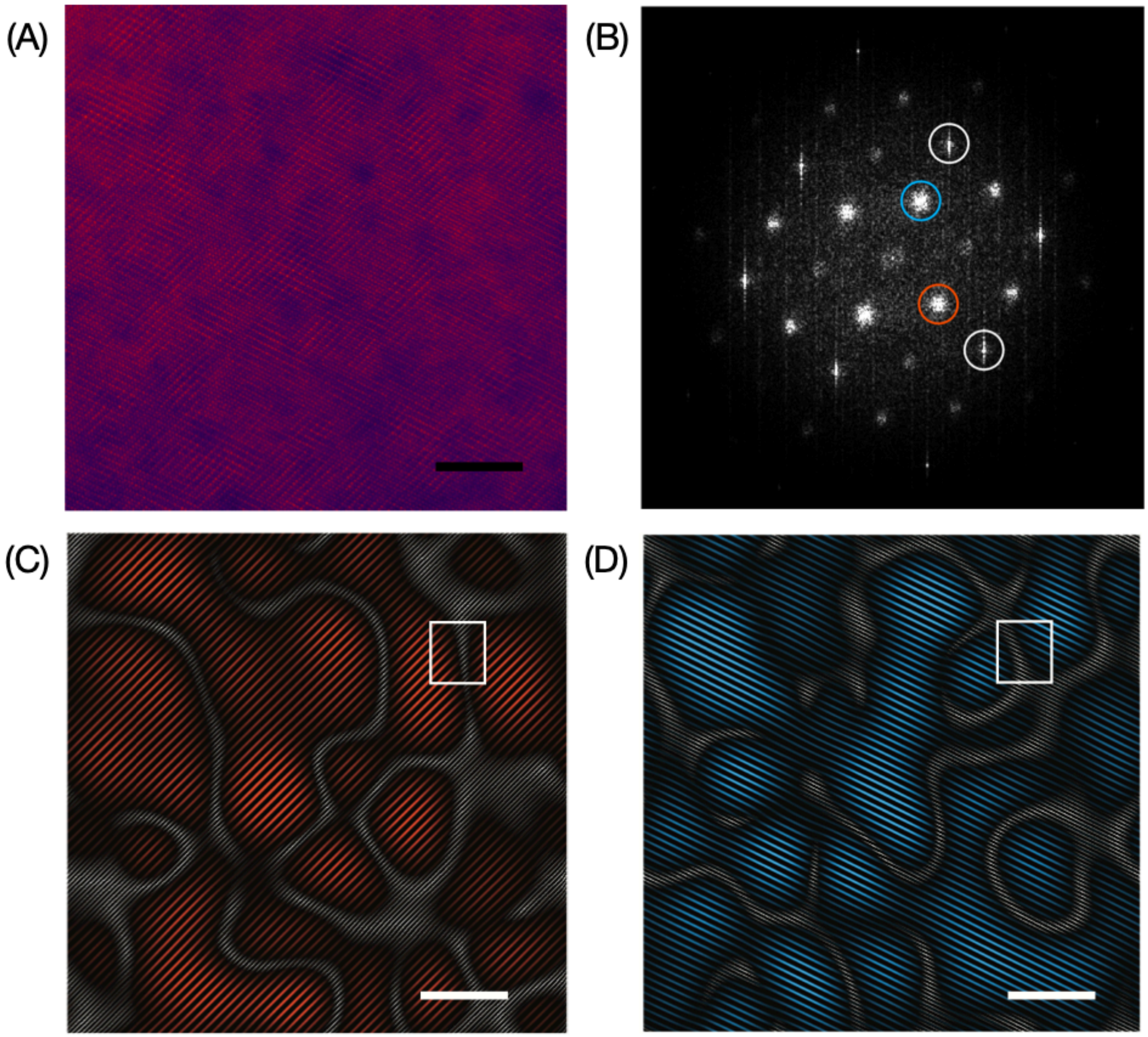

Supplementary Figure 10. Fourier filtering of the HAADF-STEM micrograph. (A) Raw HAADF-STEM micrograph shown in **Fig. 4C**. **(B)** Amplitude of the Fourier transform of the HAADF-STEM micrograph. Spinel lattice obtained by filtering the Fourier components marked by **(C)** red and **(D)** blue circles in panel B. The spinel lattice fringes are overlaid on the parent rocksalt lattice fringes obtained by filtering the Fourier spots marked as grey circle in **Fig. 4B**. (Scale bar: 5 nm).

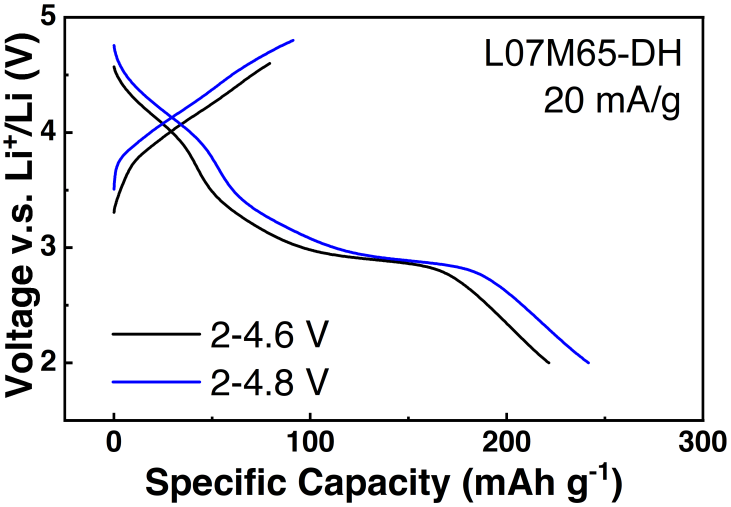

Supplementary Figure 11. Electrochemical performance of L07M65-DH with smaller particle size. Voltage profiles **L07M65-DH** for the first cycle when cycled between 2-4.8 V and 2-4.6 V at 20 mAh/g after ball milling for 20 hr at 450 rpm.

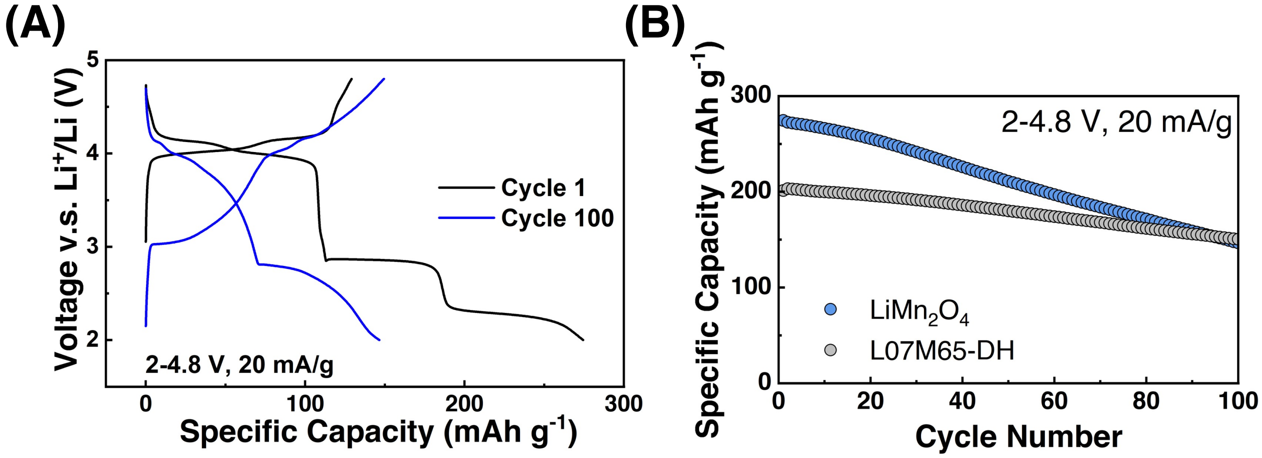

Supplementary Figure 12 Electrochemical performance of $LiMn_2O_4$. (**A**) Voltage profiles $LiMn_2O_4$ for the first cycle and 100th cycle when cycled between 2 and 4.8 V **(B)** Specific capacity retention of $LiMn_2O_4$ and L07M65-DH when cycled between 2 and 4.8 V at 20 mA/g

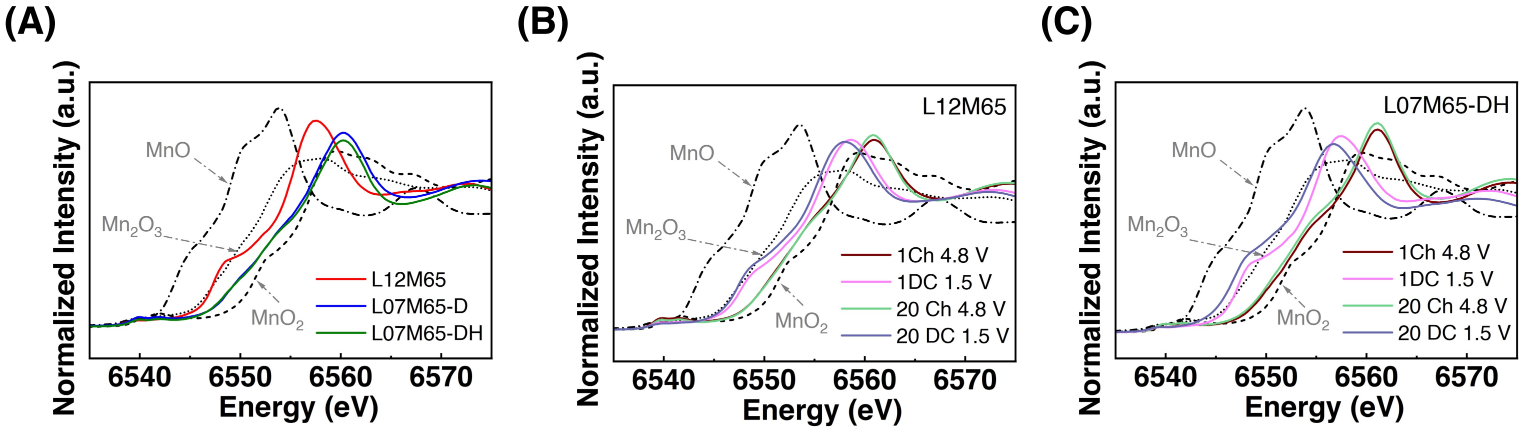

Supplementary Figure 13. The X-ray absorption near edge structure (XANES) spectra of as-synthesized L12M65 and L07M65-DH at different states of charge. Mn K-edge XANES of **(A)** L12M65 to L07M65-DH after each synthesis step. The red, blue, and green curves represent the pristine, after delithiation, and after heat-treatment samples, respectively. (**B** to **C**) (B) L12M65 and (C) L07M65-DH at different states of charge. The brown, pink, red, light green, and purple curves represent the first charge to 4.8 V (1Ch4.8V), first discharge to 1.5 V (1DC1.5V), 20th charge to 4.8 V (20Ch4.8V) and 20th discharge to 1.5 V (20DC1.5V), respectively.

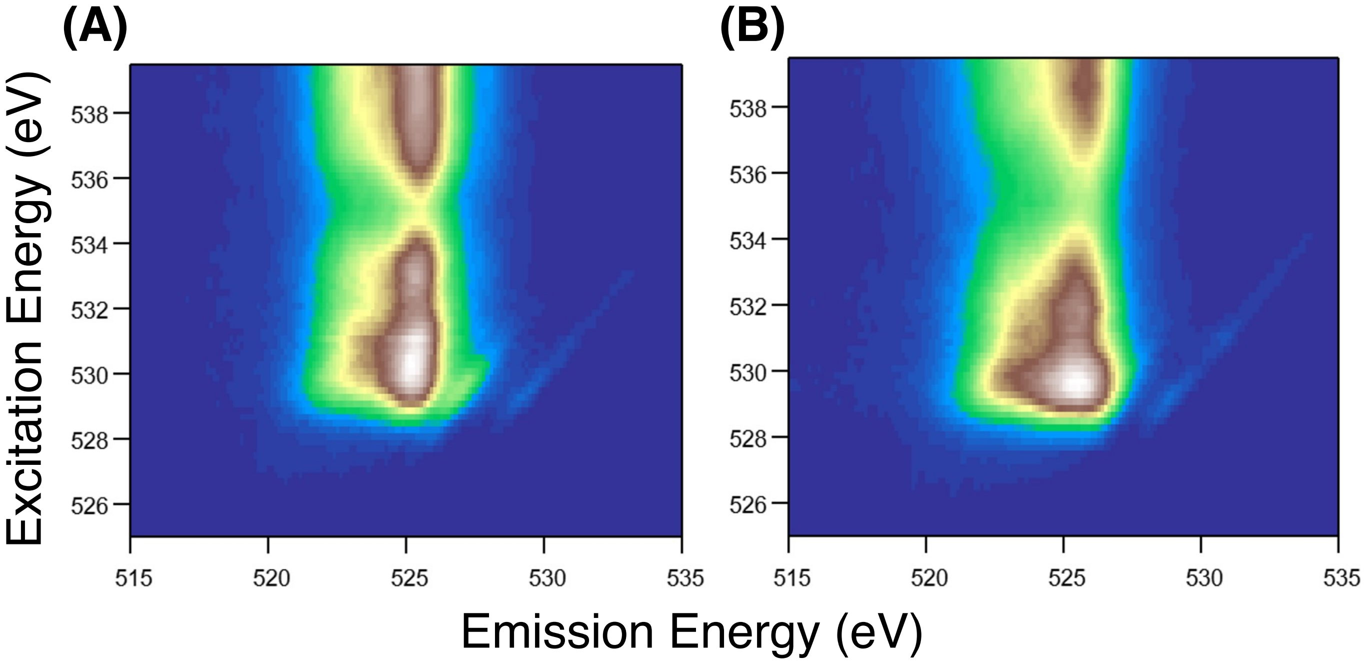

Supplementary Figure 14. *Ex-situ* O K-edge mRIXS spectra of L07M65-DH. O K-edge mRIXS of the L07M65-DH sample at (**A** to **B**) (A) Pristine L07M65-DH (B) the 1st charge to 4.8 V (1C4.8V)

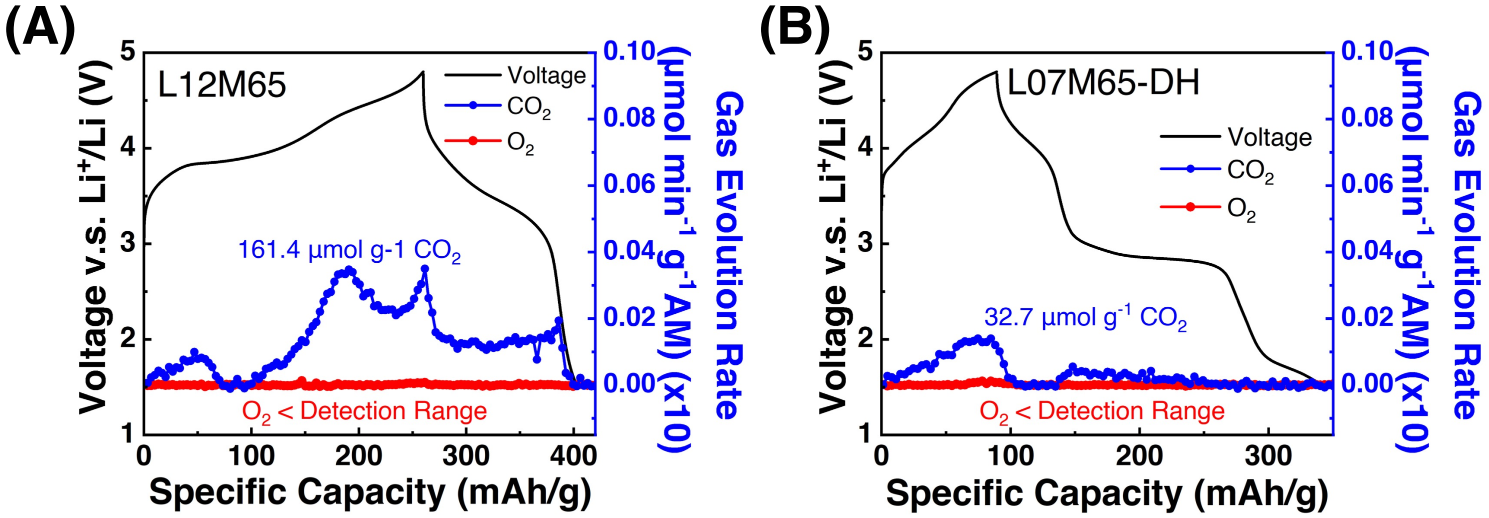

Supplementary Figure 15. Gas evolution measurements. Operando DEMS results for **(A)** L12M65 and **(B)** L07M65-DH. Voltage profile, amount of O_2 and CO_2 evolved during the first cycle are shown black solid lines, red dots and blue dots

References

1 Lun, Z. *et al.* Design principles for high-capacity Mn-based cation-disordered rocksalt cathodes. *Chem* **6**, 153-168 (2020).

2 Qiao, R. *et al.* High-efficiency in situ resonant inelastic x-ray scattering (iRIXS) endstation at the Advanced Light Source. *Review of Scientific Instruments* **88**, 033106 (2017).

3 Yang, W. & Devereaux, T. P. Anionic and cationic redox and interfaces in batteries: Advances from soft X-ray absorption spectroscopy to resonant inelastic scattering. *Journal of Power Sources* **389**, 188-197 (2018).

4 Cai, Z. *et al.* Realizing continuous cation order-to-disorder tuning in a class of high-energy spinel-type Li-ion cathodes. *Matter* **4**, 3897-3916 (2021).

5 Huang, J. *et al.* Non-topotactic reactions enable high rate capability in Li-rich cathode materials. *Nature Energy* **6**, 706-714 (2021).

6 Wu, J. *et al.* Fingerprint oxygen redox reactions in batteries through high-efficiency mapping of resonant inelastic X-ray scattering. *Condensed Matter* **4**, 5 (2019).

7 Crafton, M. J. *et al.* Dialing in the Voltage Window: Reconciling Interfacial Degradation and Performance Decay for Cation-Disordered Rocksalt Cathodes. *Journal of The Electrochemical Society* (2024).

8 Yabuuchi, N. *et al.* Origin of stabilization and destabilization in solid-state redox reaction of oxide ions for lithium-ion batteries. *Nature communications* **7**, 13814 (2016).

9 Zhang, Y. *et al.* Investigating particle size-dependent redox kinetics and charge distribution in disordered rocksalt cathodes. *Advanced Functional Materials* **32**, 2110502 (2022).

10 House, R. A. *et al.* Lithium manganese oxyfluoride as a new cathode material exhibiting oxygen redox. *Energy & Environmental Science* **11**, 926-932 (2018).

11 Lee, J. *et al.* Reversible Mn(2+)/Mn(4+) double redox in lithium-excess cathode materials. *Nature* **556**, 185-190 (2018). https://doi.org:10.1038/s41586-018-0015-4

12 Cambaz, M. A. *et al.* Design and Tuning of the Electrochemical Properties of Vanadium-Based Cation-Disordered Rock-Salt Oxide Positive Electrode Material for Lithium-Ion Batteries. *ACS Appl Mater Interfaces* **11**, 39848-39858 (2019). https://doi.org:10.1021/acsami.9b12566

13 Freire, M. *et al.* A new active Li-Mn-O compound for high energy density Li-ion batteries. *Nat Mater* **15**, 173-177 (2016). https://doi.org:10.1038/nmat4479
